# Stimulation of OGG1 Enhances Oxidative DNA Damage Repair and Protects Against Acute Liver Failure by Acetaminophen

**DOI:** 10.1002/advs.77956

**Published:** 2026-09-27

**Authors:** Zhenjun Zhao, Rahul Upadhyay, Alice Eddershaw, Chenchen Wang, Yudong Zhao, Olov Wallner, Jinhye Ryu, Nayere Taebnia, Heather Gildie, Emma Scaletti‐Hutchinson, Jonathan R. Davies, Nicole Ziegler, Andreas Krämer, Samantha C. Robinson, Marek Varga, Karolina Singerova, Zuzanna Szaruga, Elisée Wiita, İrşil Güneş, Sheila S. David, Stefan Knapp, Aimo Kannt, Miguel de Vega, Pål Stenmark, Volker M Lauschke, Maurice Michel

**Affiliations:** ^1^ Department of Liver Surgery Renji Hospital School of Medicine Shanghai Jiao Tong University Shanghai China; ^2^ Science for Life Laboratory Department of Oncology‐Pathology Karolinska Institutet Solna Sweden; ^3^ Center for Molecular Medicine Department of Oncology‐Pathology Karolinska Institute and Karolinska Hospital Stockholm Sweden; ^4^ Department of Physiology and Pharmacology Karolinska Institutet Stockholm Sweden; ^5^ Department of Biochemistry and Biophysics Stockholm University Stockholm Sweden; ^6^ Fraunhofer Institute for Translational Medicine and Pharmacology (ITMP) Frankfurt am Main Germany; ^7^ Institute of Pharmaceutical Chemistry & Structural Genomics Consortium (SGC) Goethe University Frankfurt Germany; ^8^ Department of Chemistry and Chemistry and Chemical Biology Graduate Program University of California Davis California USA; ^9^ Institute of Clinical Pharmacology Goethe University Frankfurt am Main Germany; ^10^ Centro de Biología Molecular Severo Ochoa (CSIC‐UAM) Madrid Spain; ^11^ Center for Molecular Medicine Karolinska Institutet and University Hospital Stockholm Sweden; ^12^ Dr. Margarete Fischer‐Bosch Institute of Clinical Pharmacology (IKP) Stuttgart Germany; ^13^ University of Tübingen Tübingen Germany; ^14^ Department of Pharmacy The Second Xiangya Hospital Central South University Changsha China

**Keywords:** acetaminophen, acute liver failure, DNA repair, OGG1, organocatalytic switches

## Abstract

Acetaminophen‐induced acute liver failure is characterized by reactive metabolite formation, resulting in profound oxidative stress and mitochondrial dysfunction, leading to oxidative DNA damage and loss of hepatocyte viability. While oxidative stress is well recognized as a central pathogenic driver, the contribution of DNA damage resolution pathways to hepatocyte survival remains poorly defined. Here we report the development of a small molecule, CMM‐98, that installs a robust AP‐site processing function in the DNA glycosylase OGG1 by binding the active site. Acting on the Schiff base intermediate, the molecule effectively rewires the enzyme's catalytic outcome under oxidative stress, boosting AP site turnover by 57‐fold. Using cellular and in vivo models of acetaminophen‐induced liver failure, we demonstrate that OGG1 catalysis manipulated by CMM‐98 reduces oxidative DNA damage burden, promotes the resolution of oxidative DNA lesions, and ameliorates hepatocyte injury. These findings identify DNA repair capacity as a modifiable determinant of acute liver injury outcome and establish chemical switching of OGG1 as a strategy to support hepatocyte resilience under extreme oxidative stress.

## Graphical abstract

Chemical switching of OGG1 by CMM‐98 accelerates oxidative DNA damage repair and enhances hepatocyte resilience during acetaminophen‐induced acute liver injury. By promoting efficient processing of OGG1‐generated repair intermediates, CMM‐98 reduces oxidative damage, preserves cellular integrity, and protects against acute liver failure, identifying DNA repair capacity as a pharmacologically modifiable determinant of liver injury outcome.

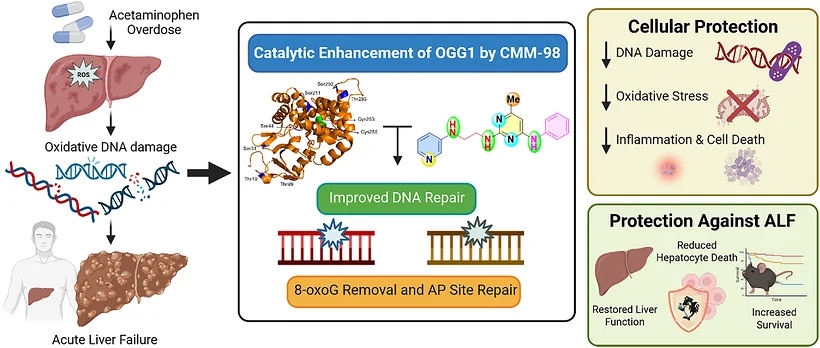

**Abbreviations**
ALF: Acute Liver Failure
ALT: Alanine Aminotransferase
AP site: Apurinic/Apyrimidinic Site
APAP: Acetaminophen (Paracetamol)
APE1: Apurinic/Apyrimidinic Endonuclease 1
AST: Aspartate Aminotransferase
BER: Base Excision Repair
CETSA: Cellular Thermal Shift Assay
CI95%: confidence interval 95
DSF: Differential Scanning Fluorimetry
DHE: Dihydroethidium
DNA: Deoxyribonucleic Acid
EDO: Ethylene Glycol Oligomer (crystallography additive)
EMSA: Electrophoretic Mobility Shift Assay
Fpg: Formamidopyrimidine DNA Glycosylase
GFP: Green Fluorescent Protein
H_2_O_2_
: Hydrogen Peroxide
HLM: Human Liver Microsomes
IF: Immunofluorescence
IHC: Immunohistochemistry
Ki67 (MKI67): Marker of Proliferation Ki‐67
LC–MS: Liquid Chromatography–Mass Spectrometry
Met‐ID: Metabolite Identification
MLM: Mouse Liver Microsomes
OGG1: 8‐Oxoguanine DNA Glycosylase 1
ORCA: Organocatalytic Switch
PAGE: Polyacrylamide Gel Electrophoresis
PNKP1: Polynucleotide Kinase/Phosphatase 1
PUA: 3′‐Phospho‐Unsaturated Aldehyde
ROS: Reactive Oxygen Species
SAR: Structure–Activity Relationship
TEM: Transmission Electron Microscopy
T_m_
: Thermal Shift
TUNEL: Terminal Deoxynucleotidyl Transferase dUTP Nick End Labeling
UBER: Universal Base Excision Repair Probe
UDG: Uracil‐DNA Glycosylase

## 1 Introduction

Acute liver failure (ALF) is a life‐threatening clinical syndrome characterized by rapid hepatocyte loss, metabolic collapse, and high short‐term mortality [1]. APAP (acetaminophen) toxicity is one of the most common causes of drug poisoning and ALF worldwide [2], particularly in high‐income countries where it is widely available over the counter. In the USA alone, APAP toxicity accounts for ∼82 000 emergency department visits, ∼38 000 hospital admissions, and ∼480 deaths annually, with estimated healthcare costs exceeding US$1 billion per year, highlighting its major clinical, public health, and socioeconomic burden [3].

Mitochondrial dysfunction and excessive production of reactive oxygen species (ROS) are drivers across diverse origins of ALF [4]. At the mitochondria, changes in permeability, impaired electron transport, and depletion of antioxidant defenses, including glutathione and manganese superoxide dismutase, drive devastating oxidative stress, which is now recognized as a central determinant of hepatocyte fate during acute injury [5].

In APAP‐induced liver injury, oxidative stress is largely driven by formation of the reactive metabolite *N*‐acetyl‐p‐benzoquinone imine (NAPQI), generated through cytochrome P450‐mediated metabolism [6]. During overdose, glutathione depletion permits NAPQI accumulation, leading to mitochondrial protein adduct formation, excessive ROS generation, and downstream oxidative DNA damage.

Excessive ROS production in ALF leads to lipid peroxidation and protein damage, as well as widespread oxidative DNA lesions in both nuclear and mitochondrial genomes [7]. Among these, oxidized purines such as 8‐oxo‐7,8‐dihydroguanine (8‐oxoG) accumulate rapidly and may interfere with transcription, replication, telomere length maintenance, genome integrity, and mitochondrial function [8, 9, 10]. Failure to resolve oxidative DNA damage has been associated with hepatocyte necrosis and poor clinical outcome, particularly in toxin‐induced liver failure [11]. Despite this, DNA damage resolution pathways have received comparatively little attention in the context of ALF, where therapeutic strategies have largely focused on limiting upstream toxic, metabolic, or inflammatory insults [1, 12].

Base excision repair (BER) represents the primary pathway responsible for the removal of oxidative DNA lesions. 8‐oxoG DNA glycosylase 1 (OGG1) plays a central role in this process by excising oxidized guanine bases and initiating repair at abasic (AP) sites [8, 13]. While OGG1 rapidly excises oxidized bases, its weak intrinsic AP‐site lyase activity is negligible in cells, resulting in the accumulation of OGG1‐bound AP‐site intermediates under conditions of oxidative stress [13]. Emerging evidence suggests that such stalled repair complexes engage transcriptional and inflammatory signaling pathways [10, 12, 14, 15]. Therefore, the removal of AP sites from OGG1 and cleavage through APE1 becomes rate‐limiting during periods of acute oxidative stress [16]. As a result, BER capacity may become saturated in ALF, allowing oxidative lesions and toxic intermediates to persist.

In the past, mitochondrial OGG1 protein isoforms as well as enzymatic enhancement through post‐translational modifications have been shown to protect against high‐fat diets, obesity [17, 18], and neuroinflammation, particularly in Alzheimer‘s Disease [19, 20]. In addition, recent work has begun to challenge the view of DNA repair enzymes as static housekeeping factors, instead revealing a degree of catalytic plasticity that can be chemically exploited [15, 16, 21, 22, 23, 24]. Small‐molecule modulators called organocatalytic switches (ORCAs) have been shown to alter the activity profile of OGG1, including the release from long‐lasting AP site intermediates, enhancing OGG1 substrate turnover [25, 26]. This mechanism suggests that repair outcome may be tunable rather than only regulated up or down through classical inhibitors and activators [14, 27, 28, 29, 30]. However, whether such modulation can be harnessed to deliberately redirect DNA repair chemistry and influence cell survival during acute organ failure has remained unexplored.

Here, we address this question by applying the concept of chemical switching to OGG1 in models of APAP‐induced acute liver injury [26]. We show that ORCAs can install a robust AP‐site processing function in OGG1, effectively rewiring its catalytic outcome under conditions of extreme oxidative stress. Through combined chemical, biochemical, and structural investigations, we demonstrate that CMM‐98 promotes an otherwise negligible AP lyase activity. Using cellular and in vivo models of APAP‐induced liver failure, we demonstrate that ORCA‐mediated acceleration of OGG1 catalysis reduces oxidative DNA damage burden and enhances hepatocyte stress tolerance. Together, these findings identify DNA repair capacity as a critical element of acute liver injury outcome and demonstrate that targeting OGG1 using ORCAs serves as a strategy to maintain hepatocyte health under oxidative collapse.

## 2 Results

### 2.1 Development of CMM‐98, A Next‐Generation OGG1‐ORCA

Previously, we observed first‐generation ORCAs assisting OGG1 in resolving AP sites in two distinct manners, either via a PNKP1‐dependent or an APE1‐dependent pathway [16, 31, 34]. Subsequent mechanistic analysis revealed that the unique PNKP1‐dependent OGG1 mechanism in the presence of TH10785 arises from a reorganization of the OGG1‐AP‐site Schiff base intermediate, relieving a conformational constraint imposed by Asp268 on the DNA backbone [35]. In addition, when screening a library of OGG1‐ORCAs and other DNA repair‐enhancing molecules [20, 36, 37] in a patient‐derived model of metabolic‐dysfunction associated steatohepatitis (MASH), we observed antifibrotic effects, enhanced DNA repair, and dampened inflammatory programs for both OGG1 AP lyase functions [32, 38]. Furthermore, targeted proteomics revealed a rewiring of cellular pathways, indicating hepatocyte regeneration.

**SCHEME 1:**
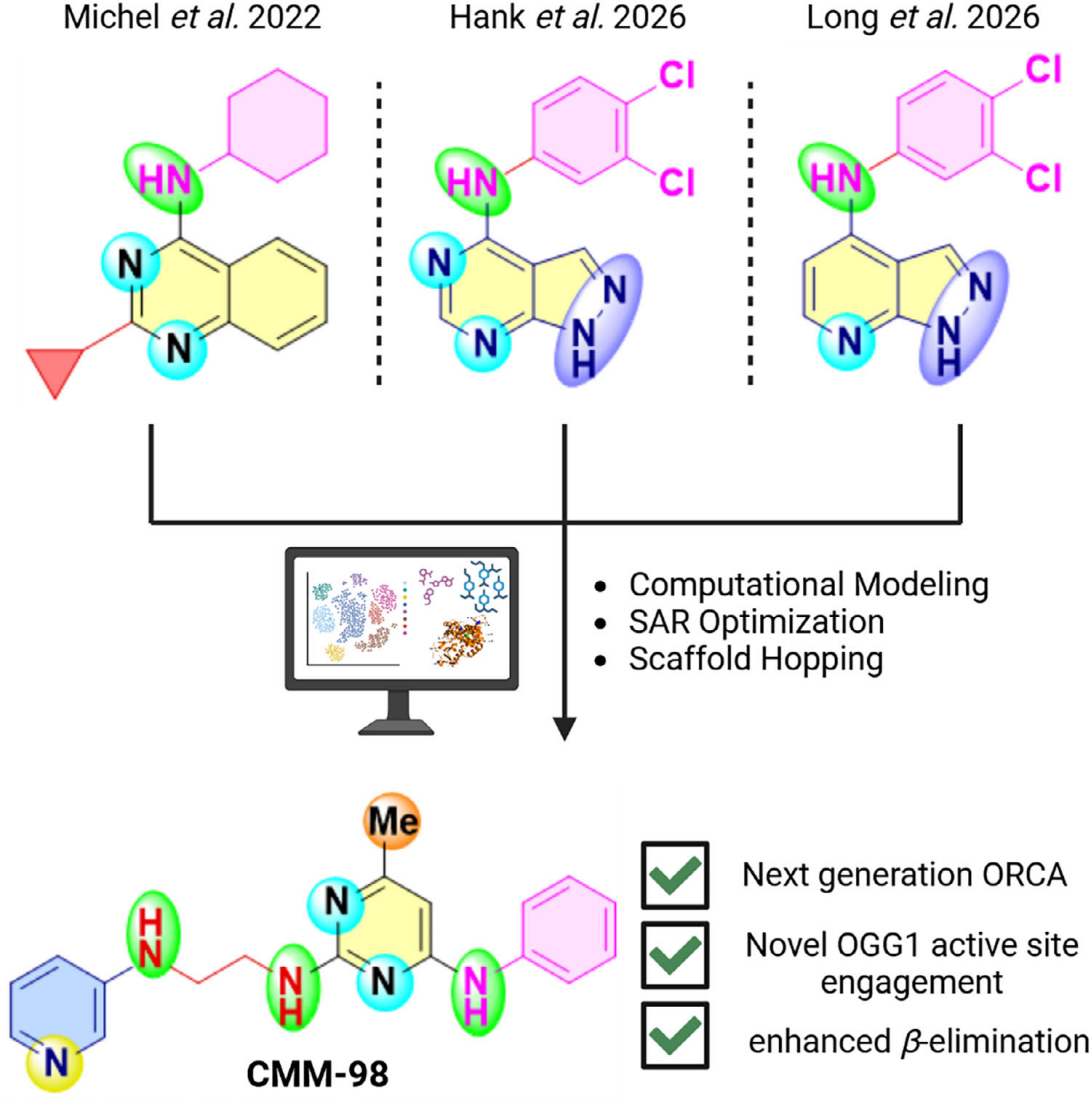
Rationale for the development of CMM‐98: Previously, we reported a distinct chemical series based on the original ORCA TH10785 and nucleobase space [16, 28, 29, 31, 32, 33]. Here, we use computational approaches, SAR optimization, and a scaffold hopping approach to discover CMM‐98, which demonstrates new interactions within the OGG1 active site while maintaining reactivity on the intermediate Schiff Base. The color code of the molecules indicates corresponding positions among the different chemical series. Pink–deep pocket of OGG1; green spheres–H‐bonding to Gly42; pale yellow–π‐stacking to Phe319; light blue spheres–nitrogen atoms for proton abstraction; red–unexplored vector within TH10785 exploited for CMM‐98; dark blue spheres–mitochondria targeting moieties; orange sphere–vector replacing the second annealed ring (pale yellow) in previous chemical series.

Based on previous observations that ORCAs undergo substantial hepatic metabolism [34], we sought to optimize their physicochemical and pharmacokinetic properties to enhance functional activity in the liver. Given that the liver is highly susceptible to damage associated with oxidative stress, such optimization may enable more effective modulation of OGG1 activity in hepatocytes. Combining accumulation in the liver with an advancement in physicochemical properties compared to the challenging parameters of TH10785 [34], it may be possible to support hepatocyte function in chronic as well as acute liver injuries. To maintain the activity of the quinazoline scaffold of TH10785, we employed a fragment‐growing strategy, starting from a pyrimidine ring previously reported in nucleobase‐inspired ORCAs (Scheme 1) [28, 29, 31, 32]. Growing the molecule into more complex chemical space required systematic modification of the pyrimidine core at both the 2‐ and 4‐positions, which are equivalent to the cyclopropyl (red) and cyclohexyl‐amine (pink) substituents in TH10785 (Scheme 1). Through iterative optimization, we identified CMM‐98 as a lead compound, exhibiting an AC_50_ of 0.038 µM on hOGG1 (CI95% 0.022–0.066), inducing thermal stabilization of human OGG1 (ΔT_m_ = 1.1 K at 200 µM). The AC_50_ on mouse OGG1 was 0.083 µM (CI95% 0.014–0.151)

**FIGURE 1:**
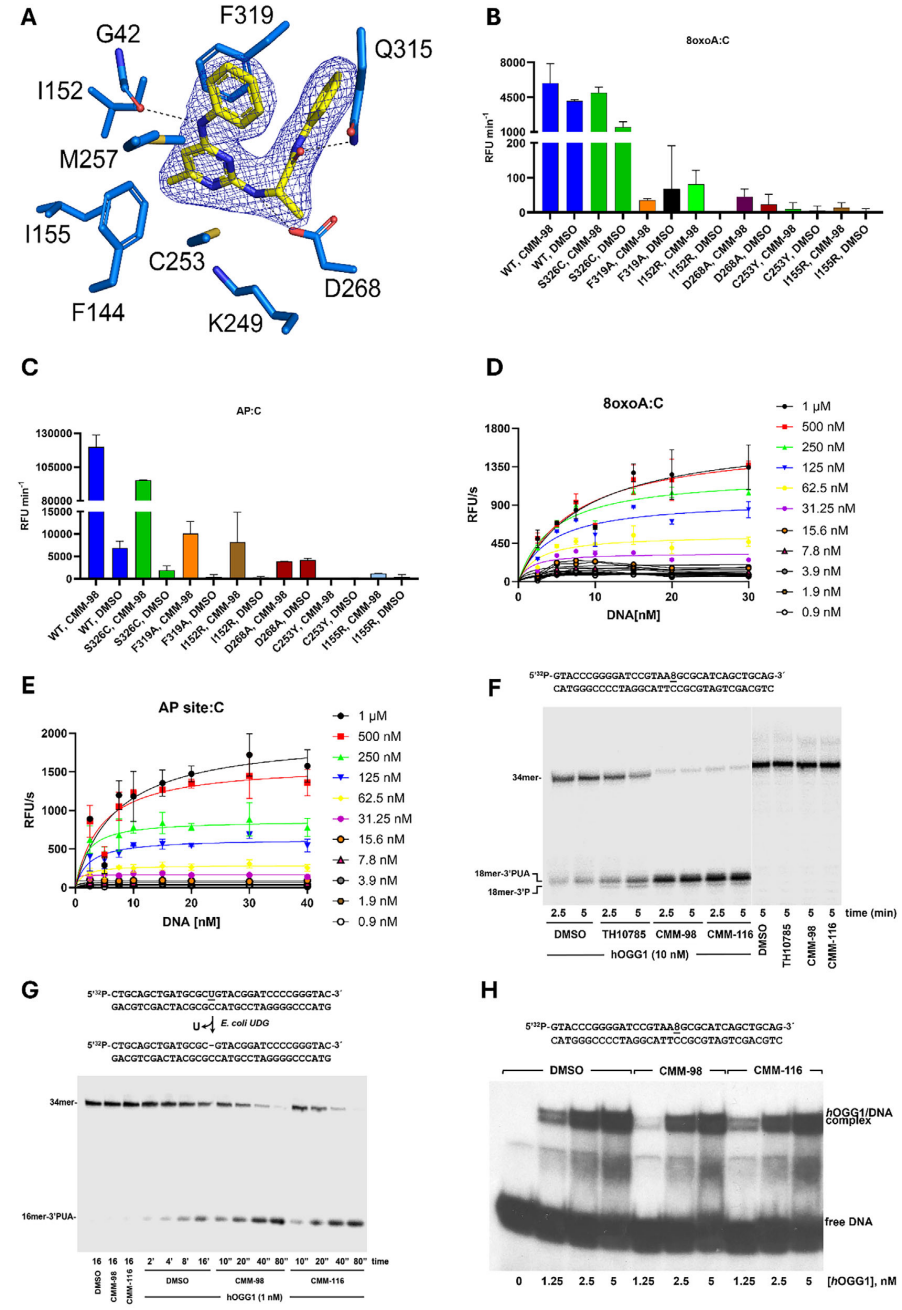
Development and mechanistic characterization of CMM‐98 as an OGG1‐ORCA: (A) Crystal structure of murine OGG1 in complex with a CMM‐98 analog (CMM‐115), highlighting active‐site interactions. The ligand (carbon–yellow, nitrogen—blue, oxygen—red) is shown, forming hydrogen‐bond interactions with Gly42, Asp268, and Gln315, and hydrophobic interactions with residues including Phe319, Phe144, Ile152, and Met257. The 2Fo‐Fc electron density map around CMM‐115 is contoured at 1.0 σ (blue mesh), and the Fo‐Fc electron density maps are contoured at −3.5 σ (red mesh) and +3.5 σ (green mesh). The binding supports productive active‐site engagement and catalytic modulation. (B) Assessment of active site and proximal mutations (see A) of OGG1 against the substrate 8‐oxoA:C in the presence of 8 µM CMM‐98 supports active site binding for catalysis. All proteins were assayed at 10 nM, apart from Ser326Cys at 50 nM, with 10 nM substrate duplex Oligo, RFU min^−1^–relative fluorescence units per min. (C) The same conditions were used against substrate AP site:C generated from U:C by 1 nM UNG2. (D) Kinetic analysis of OGG1 activity on 8‐oxoA:C in the presence of ORCAs. Concentration‐dependent stimulation of catalytic activity is shown, with CMM‐98 displaying enhanced activation. Representative dataset shown with mean ± [SEM/SD]. (E) Kinetic analysis of OGG1 activity on an AP site:C in the presence of CMM‐98. Substrate generated from U:C by 1 nM UNG2. Representative dataset shown with mean ± [SEM/SD]. (F) Analysis of the effect of CMM‐98 and CMM‐116 on hOGG1 activity. Upper panel: scheme of the substrate. The assay was performed by incubating 1 nM of the [^32^P]5’‐labeled 8oxoG‐containing substrate with 10 nM hOGG1 (where indicated) and either 10% DMSO or 6 µM of the indicated compound. Samples were analyzed by 7 M urea‐20% PAGE and autoradiography. The position of products is indicated. The figure is a composite image made from different parts of the same gel. (G) Analysis of the effect of CMM‐98 and CMM‐116 on hOGG1 activity. Upper panel: scheme of the substrate. The assay was performed by incubating 1 nM of the [^32^P]5’‐labeled uridin‐containing substrate, previously treated with 0.2 U of E. coli UDG to obtain a natural AP site, with 1 nM hOGG1 (where indicated) and either 10% DMSO or 6 µM of the indicated compound. Samples were analyzed by 7 M urea‐20% PAGE and autoradiography. The position of products is indicated. (H) Analysis of the effect of CMM‐98 and CMM‐116 on the DNA‐binding ability of hOGG1. The assay was performed by incubating 1 nM of the [^32^P]5’‐labeled 8‐oxodG:C‐containing substrate with the indicated concentrations of hOGG1, in the presence of either 10% DMSO or 6 µM of the indicated compound. Protein/DNA complexes were resolved in non‐denaturing 6% polyacrylamide gels and subjected to subsequent autoradiography.

Since ORCAs assist OGG1 in catalysis using a basic moiety within the molecular scaffold [32, 34], while also exploiting interactions that are employed by the enzyme to recognize 8‐oxoG in DNA [22], we first needed to confirm active‐site engagement. Guiding our optimization, we used structure‐based design, visualizing ligand engagement in the crystal structure of murine OGG1 in complex with the analog CMM‐115 at 2.40 Å resolution (Table S1). Although CMM‐115 contains additional substituents relative to CMM‐98, both compounds share the same active pharmacophore and exhibit comparable dependence on key active‐site residues. CMM‐115 was therefore used as a crystallographic surrogate due to superior crystallization properties. Clear and unambiguous electron density corresponding to CMM‐115 was observed within the active site (Figure 1A). Mouse OGG1 was used for crystallographic studies because its active‐site architecture is identical to that of human OGG1 and it has superior crystallization properties.

CMM‐115 is anchored by a network of hydrogen bonds involving the secondary amine adjacent to the phenyl ring and the backbone carbonyl oxygen of Gly42, the exocyclic secondary amine and the side‐chain carboxylate of Asp268, and the urea carbonyl oxygen and the side‐chain amide of Gln315. In addition, ligand binding is stabilized by a π‐stacking interaction with Phe319 and extensive hydrophobic contacts with Phe144, Ile152, Ile155, Lys249, Cys253, Met257, Pro266, and Val271 (Figure 1A).

Superposition of the mOGG1‐CMM‐115 structure with the previously reported hOGG1‐TH10785 complex (PDB ID: 7AYY) reveals overall structural similarity of the protein backbone (Figure S1A). Both ligands occupy the active‐site pocket; however, their core scaffolds adopt distinct orientations. While TH10785 engages Asp268 and Gly42, it does not form the Gln315 interaction observed for CMM‐115. Moreover, the side chains of Ile152, Asp268, Lys249, and Phe319 adopt markedly different conformations between the two structures (Figure S1B), consistent with ligand‐induced rearrangement of the catalytic pocket. Overall, this study confirmed the orientation of the central pyrimidine ring, while CMM‐115 and CMM‐98 expanded into new areas of the OGG1 active site.

While our X‐ray co‐crystal structure represents a static snapshot of an interaction without DNA [22], we sought to corroborate the observation through activity assessment of OGG1 mutants. Thus, we evaluated CMM‐98 across a panel of OGG1 variants, including active site and distant mutations, using a previously reported fluorophore‐quencher assay [31]. Measuring activity of the mutants against the more stable substrate 8‐oxoA:C, we observed no difference between DMSO and CMM‐98 for Phe319Ala, Cys253Tyr, Asp268Ala, Ile152Arg, and Ile155Arg, confirming previous findings [34, 35]. Activity was markedly enhanced for wild‐type OGG1 and the Ser326Cys variant, which lies proximal to but outside of the catalytic pocket (Figure 1B). When assessing the same mutants and CMM‐98 against an AP site opposite cytosine generated from uracil using UNG2, we observed strong activation of wild‐type OGG1 and Ser326Cys, as well as low‐level activation of Phe319Ala and Ile152Arg (Figure 1C). In contrast to TH10785, CMM‐98, which engages the active site via Asp268, did not accelerate the rate of the mutant Asp268Ala [35, 39]. These data are in accordance with previous findings of a measurable AP‐lyase activity of the Ile152Arg, Phe319Ala, and Asp268Ala mutants with TH10785 [16, 35]. The comparable increased activation and higher efficacy on AP sites, together with the activity profile and the X‐ray co‐crystal structure, support a productive binding mode of ORCAs that depends on an intact active‐site architecture of OGG1.

Using the fluorophore‐quencher assay, we next assessed the functional consequences of CMM‐98 on OGG1 catalysis using duplex DNA substrates containing either 8‐oxoA:C or AP:C base pairs (Figure 1D,E). Saturating concentrations of substrate were used to evaluate the effect of CMM‐98 on substrate turnover. Activity on both substrates was stimulated, with the AP lyase activity peaking at 57‐fold enhancement over the DMSO control at 1 µM concentration (Figure 1E). In contrast, 8‐oxoA excision and AP site incision, as monitored by the 8‐oxoA substrate, indicated a competition of CMM‐98 binding with the 8‐oxoA substrate at higher concentrations, an effect previously reported (Figure 1D) [16, 26, 32]. Importantly, the use of 8‐oxoA as a substrate rather than 8‐oxoG did not affect the rate of the reaction as reported earlier [31].

As compound matter might interact in various ways with the fluorophore‐quencher assay, an assay technology with orthogonal readout may be used to exclude pan‐assay interference and to confirm true efficacy of ORCAs on OGG1 catalysis. To further determine the precise mode of incision, i.e., a *β*‐ or *β,δ*‐elimination, we performed denaturing PAGE analysis using ^32^P‐labelled DNA substrates containing 8‐oxoG and an AP site generated from uracil using *E.coli* UDG (Figure 1F,G). Using both CMM‐98 and minimal structure CMM‐116 (Supporting Information, Small molecule Characterization), we confirmed the stimulation of the canonical *β*‐AP lyase activity, generating the characteristic 3′‐PUA DNA end from both AP sites and 8‐oxoG substrates. As previously observed, the reaction profile of TH10785 against 8‐oxoG substrates further generated a 3’P, by a *β,δ*‐elimination chemistry in OGG1 [16]. Neither CMM‐98 nor CMM‐116 exerted an effect on the two substrates in the absence of OGG1, ruling out an unspecific cleavage of DNA. In addition, we assessed whether the compounds influenced the DNA binding ability of OGG1 (Figure 1H). In the presence of an 8‐oxoG substrate and dependent on compound activity, less DNA was observed in complex with the protein, indicating the stimulated release from the intermediate Schiff Base moiety. Together, these experiments indicate that CMM‐98 binds the active site of OGG1 and specifically stimulates a *β*‐AP lyase activity of the enzyme.

To further evaluate the developability of CMM‐98, we performed an initial assessment of physicochemical and ADME properties. The compound exhibited improved aqueous solubility (28 µM) compared to TH10785 (6 µM) [34]. In vitro metabolic stability studies revealed high intrinsic clearance in both human and mouse liver microsomes (HLM Clint = 336.0 µL min^−^^1^ mg^−^^1^; MLM Clint = 510 µL min^−^^1^ mg^−^^1^), indicating that the compound is susceptible to rapid hepatic metabolism. These data suggest that hepatic metabolism may limit exposure to the parent compound. The identity, pharmacological activity, and tissue distribution of the resulting metabolites were not investigated and therefore cannot be inferred from the microsomal stability assay.

Importantly, selectivity profiling across a panel of BER enzymes and a wider kinase panel revealed no detectable off‐target activity, supporting a high degree of specificity for OGG1 (Table S2 and S3). Together, these results establish CMM‐98 as a selective OGG1‐ORCA that engages the active site to enhance AP‐site processing. Initial ADME profiling indicated hepatic metabolism, consistent with our goal of accumulation in the liver. These findings provided a foundation for further in vivo evaluation of OGG1‐directed repair modulation.

### 2.2 CMM‐98 Enhances APE1‐Dependent Oxidative DNA Repair and Preserves Mitochondrial Integrity

**FIGURE 2:**
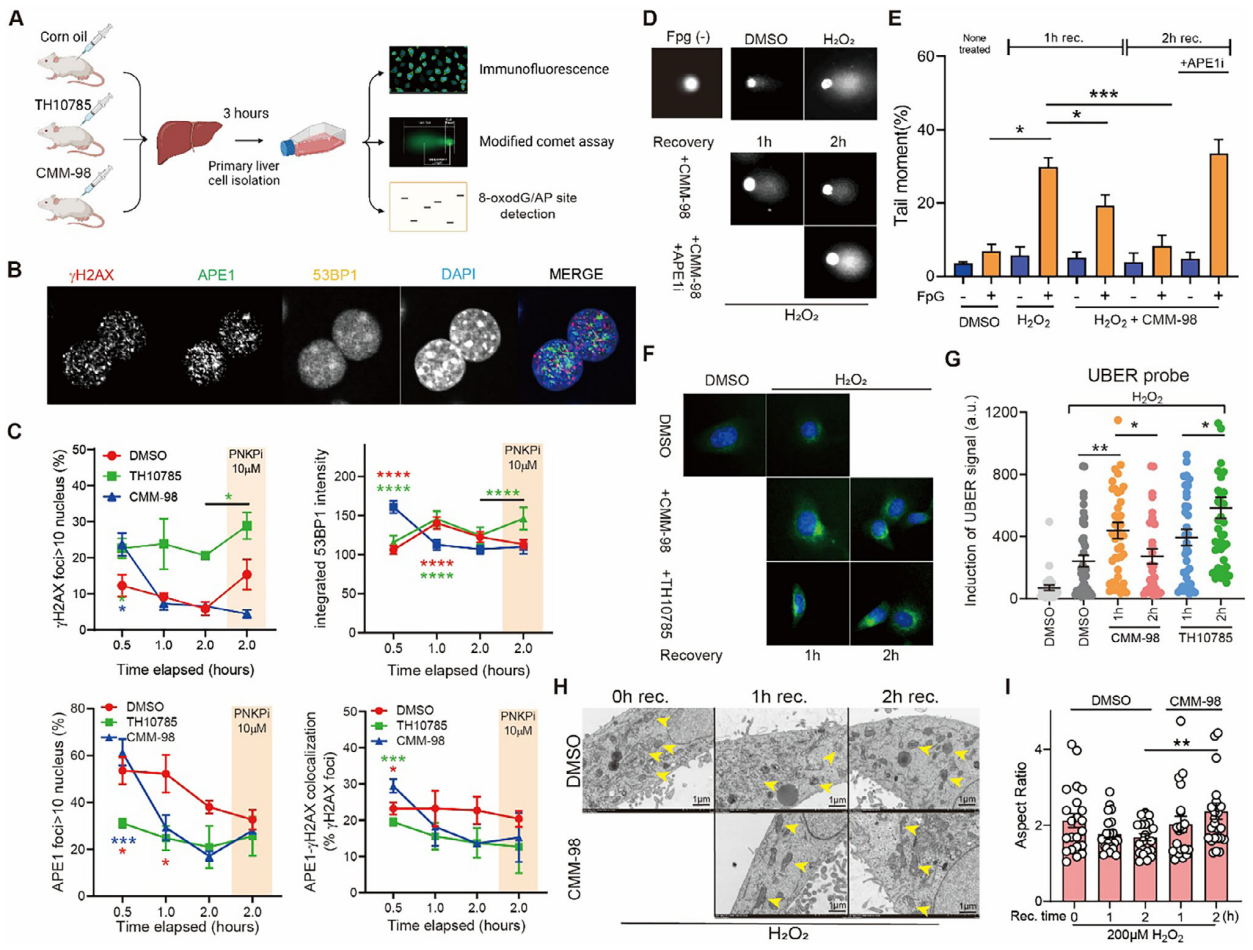
CMM‐98 attenuates oxidative DNA damage in primary hepatic cells: (A) Schematic figure showing the establishment of experiments in B–G. Briefly, mice were injected with different compounds for 8 h before primary hepatic cells were isolated. After washing away detached primary hepatic cells, cells were treated separately in immunofluorescence (B,C), modified comet assay (D,E), and labeled with UBER probe for AP site quantification (F,G). (B,C) Typical figures (B) and quantifications (C) of γH2AX foci, APE1 foci, 53BP1 foci, and the γH2AX‐APE1 colocalization in primary hepatic cells after treatment with H_2_O_2_ and recovery at the indicated time with different compounds. 20 nM CMM‐98, 10 nM TH10785 and 10 µM PNKP1 inhibitor (PNKP1) were used. (D,E) Typical figures (D) and quantifications (E) of tail moments in the modified comet assay using FpG enzyme. Primary hepatic cells were treated with H_2_O_2_ and recovered for 1 or 2 h with 20 nM CMM‐98 or 20 nM CMM‐98 + 10 µM APE1i. (F,G) Typical figures (F) and quantifications (G) of AP sites recognized by the UBER probe. Fluorescence intensity was quantified in at least 25 cells after H_2_O_2_ treatment and recovery for 1, 2 h with 20 nM CMM‐98 or 10 nM TH10785. (H‐I) Representative Images (H) and quantifications (I) of electron microscopy results showing the alterations in mitochondrial morphology after H_2_O_2_ treatment and recovery with 20 nM CMM‐98. The aspect ratio was defined as the long axis/short axis of mitochondria (n ≥ 18 in each group). For immunofluorescence and modified comet assay, at least 40 cells were quantified per biological replicate, with at least 3 independent biological replicates per group. Data are presented as mean ± SEM. Statistical significance was determined using a two‐sided Student's *t*‐test for pairwise comparisons. ns, not significant; ^*^ *p* < 0.05; ^**^ *p* < 0.01; ^***^ *p* < 0.001; ^****^ *p* < 0.0001.

Since we had previously observed signs of improved hepatocyte regeneration under stress [38], we first set out to evaluate the impact of catalytic enhancement of OGG1 on oxidative DNA repair in primary hepatocytes. We pretreated mice with CMM‐98, TH10785, or vehicle (DMSO in corn oil) prior to primary hepatocyte isolation, followed by H_2_O_2_ challenge and time‐resolved recovery analyses (Figure 2A). To first assess general DNA damage markers, we performed immunofluorescence staining, which revealed that both compounds facilitated the resolution of DNA damage‐associated foci, as indicated by dynamic changes in γH2AX and 53BP1 foci (Figure 2B,C). In the next step, we sought to validate the in vitro finding that CMM‐98, and much less so TH10785, act through *β*‐elimination, i.e., have APE1‐dependency, in cells. We indeed observed divergent repair mechanisms for CMM‐98 and TH10785. TH10785‐mediated reduction of γH2AX and 53BP1 foci was reversed by PNKP1 inhibition, demonstrating dependence on PNKP1 for strand break processing (Figure 2C, upper panels). Furthermore, whereas TH10785 maintained persistently low APE1 foci, consistent with a reduced APE1‐dependent pathway, CMM‐98‐driven repair was associated with rapid clearance of APE1 foci during recovery (Figure 2C, lower panel), indicating accelerated turnover of AP intermediates through APE1‐dependent BER. To confirm the distinction, we then assessed γH2AX‐APE1 colocalization, where CMM‐98 markedly enhanced early colocalization followed by rapid resolution, reflecting increased BER flux, while TH10785 failed to induce comparable APE1 engagement (Figure 2C).

Having confirmed DNA lesion flux along the distinct pathways, we interrogated how ORCAs facilitate the processing of oxidized lesions via the BER pathway. To this end, we performed a modified Comet assay in primary hepatocytes, incorporating digestion using the broadly active bacterial DNA glycosylase Fpg [40]. This served to detect 8‐oxoG lesions and at the same time resulted in DNA strand incision through robust AP‐lyase activity. The results demonstrated that CMM‐98 significantly reduced tail moments at 1 and 2 h of recovery (Figure 2D,E), indicating accelerated oxidative lesion repair. This effect was successfully abolished by APE1 inhibition, confirming APE1 dependency of CMM‐98‐mediated repair. In the following step, we performed live‐cell labeling of AP sites using the UBER probe [41, 42], further revealing a transient increase in AP signal at 1 h after CMM‐98 treatment, followed by rapid decline at 2 h (Figure 2F,G), consistent with enhanced generation and efficient processing of OGG1 BER intermediates. In contrast, cells treated with TH10785 exhibited persistently low AP site levels throughout recovery. Finally, using transmission electron microscopy, we assessed mitochondrial morphology in cells under oxidative stress. Using H_2_O_2_ exposure, we observed induced mitochondrial rounding and fragmentation, reflected by reduced aspect ratio, whereas additional treatment with CMM‐98 restored mitochondrial elongation and significantly increased the long‐to‐short axis ratio (Figure 2H,I). Collectively, these results demonstrate that CMM‐98 accelerates APE1‐dependent BER, promotes rapid turnover of oxidative repair intermediates, and preserves mitochondrial structural integrity in primary hepatic cells. Although mitochondrial morphology was markedly improved, our data do not directly assess mitochondrial DNA repair, and the observed structural preservation may arise from reduced overall oxidative stress following enhanced DNA repair. These findings contrast with the PNKP1‐dependent mechanism of TH10785.

### 2.3 CMM‐98 Protects Against APAP‐Induced Acute Liver Failure by Suppressing DNA Damage, Oxidative Stress, and Mitochondrial Dysfunction

**FIGURE 3:**
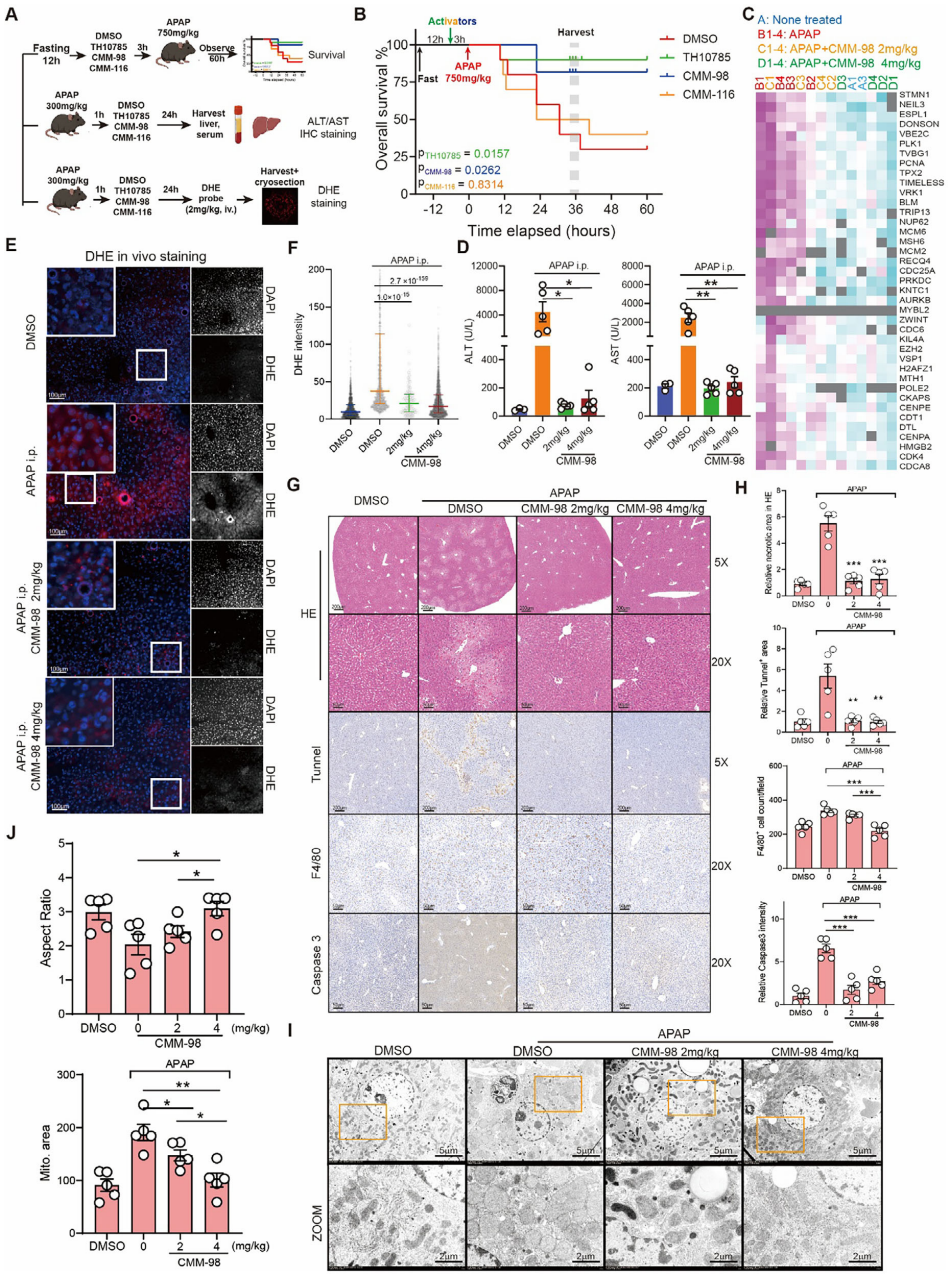
CMM‐98 rescues APAP‐induced liver damage in vivo: (A) Schematic figure showing the establishment of the biological function of CMM‐98 in vivo. Briefly, mice were administered a lethal dose of APAP in the presence or absence of the indicated compounds to assess survival. For biochemical and histological analyses, a sub‐lethal APAP model was used, and serum and liver tissues were collected to evaluate DNA damage and liver injury. For oxidative stress assessment, dihydroethidium (DHE) was administered intravenously in vivo, and liver tissues were subsequently harvested, cryo‐sectioned, and analyzed ex vivo for fluorescence imaging and quantification of superoxide levels. (B) Kaplan–Meier analysis showing the overall survival of the APAP‐induced acute liver damage model. Mice (n = 10 in each group) were fasted for 12 h and given different compounds. After 3 h, all mice were treated with 750 mg/kg APAP i.p. and followed for 48 h with a normal chow diet. Mice were given 4 mg/kg TH10785, 8 mg/kg CMM‐98, and 10 mg/kg CMM‐116, respectively. A negative control group of mice was given corn oil at 200 µL. (C) Heatmap showing the quantification of 39 distinct DNA damage markers in the mice liver tissues. Mice were treated as in (B). (D) Quantification of liver functions (ALT and AST levels) in mice treated as in (B). Blood serum was also collected after 24 h. (E,F) Typical figures (E) and quantification (F) of DHE probe signal in mouse liver samples after APAP and CMM‐98 treatment (at least 100 cells were analyzed). Mice were given CMM‐98 1 h before APAP was injected. 24 h after APAP injection, mice were intravenously injected with 100 µL of DHE probe at 5 mM. 15 min after the injection, mice were sacrificed, and liver tissues were embedded using OCT and subjected to ex vivo fluorescence imaging to assess superoxide levels. At least 100 cells per group were analyzed. Bars indicate the median with 2.5th–97.5th percentile error bars. (G‐H) Typical figures (G) and quantifications (H) of HE staining and IHC staining results (Tunnel, F4/80, and Caspase 3). Mice (n = 5 in each group) were treated with 200 mg/kg APAP or 200 mg/kg APAP with 2–4 mg/kg CMM‐98 for 24 h. (I‐J) Typical electron microscopy images (I) and quantification (J) of mitochondrial morphology. Mice (n = 5 in each group) were treated with 200 mg/kg APAP or 2–4 mg/kg CMM‐98, and mitochondrial images were acquired using electron microscopy. Aspect ratio (long axis/short axis) and mitochondrial area were manually quantified using ImageJ in (J). Data are presented as mean ± SEM unless specified. Survival curves were analyzed using the Kaplan‐Meier method with the log‐rank (Mantel‐Cox) test. All other statistical comparisons were performed using a two‐sided Student's *t*‐test. ns, not significant; ^*^ *p* < 0.05; ^**^ *p* < 0.01; ^***^ *p* < 0.001.

We hypothesized that the rapid clearance of DNA damage mediated by CMM‐98 offers the potential to alleviate acute bursts of reactive oxygen species damaging DNA. Thus, we set out to determine whether stimulation of the OGG1 AP lyase activity in hepatocytes might provide therapeutically relevant protection against ALF in vivo. To this end, we used a mouse model of APAP‐induced ALF [43, 44] and performed a comprehensive evaluation, including survival analysis, liver function tests (ALT/AST), hepatic DNA damage profiling, ROS detection, histopathology, and ultrastructural assessment (Figure 3A). Kaplan‐Meier analysis of the model revealed that APAP administration caused severe mortality, with survival rates falling below 30% within 48 h. Remarkably, when we pretreated with either TH10785 or CMM‐98, we observed a dramatically improved survival of >80%, whereas a minimal structure of CMM‐98, CMM‐116 (AC_50_ 0.147 µM (CI95% 0.131–0.165), Thermal Stabilization in DSF: [45] 1.4 K at 200 µM, solubility 64 µM, HLM Clint = 130.0 µL min^−^^1^ mg^−^^1^; MLM Clint = 276.0 µL min^−^^1^ mg^−^^1^), had no protective effect (Figure 3B), indicating functional specificity among OGG1 modulators. To more closely model a clinically relevant APAP overdose scenario, TH10785 or CMM‐98 was administered 1 h after the APAP challenge. CMM‐98 maintained similarly high survival rates, whereas survival was markedly lower in the TH10785‐treated group (Figure S2A). Importantly, however, both post‐APAP treatments improved survival compared with APAP alone.

At 24 h after APAP challenge, we harvested liver tissues and first observed that DNA damage‐associated genes were markedly enhanced, indicating upregulated DNA repair as a response to the APAP overdose (Figure 3C). The comparison to the CMM‐98‐treated cohort revealed a significant normalization of this transcriptional response and restored gene expression patterns toward baseline levels. In the next step, we assessed serum ALT and AST levels as proxies of liver damage. In alignment with the gene expression results above, we observed a sharp elevation 3–24 h after APAP exposure, while a reduction to near‐normal levels was observed after receiving CMM‐98. These results demonstrated substantial preservation of liver function (Figure 3D and Figure S2B). In addition, in vivo ROS staining using dihydroethidium (DHE) and γH2AX further showed that APAP induced robust oxidative stress in liver tissue, whereas CMM‐98 administration markedly suppressed ROS accumulation (Figure 3E,F and Figure S2C,D), supporting an antioxidative and damage‐limiting effect.

Next, we sought to corroborate these findings by histopathological analysis. Indeed, APAP‐treated livers exhibited extensive hepatocellular necrosis and increased DNA fragmentation (TUNEL staining). In comparison, CMM‐98 markedly reduced necrotic areas and DNA fragmentation (Figure 3G,H). Further, we observed pronounced macrophage infiltration (F4/80 staining) following APAP challenge, indicating an inflammatory response. A significant attenuation was observed with 4 mg kg^−^^1^ CMM‐98, while 2 mg kg^−^^1^ failed to reduce immune cell infiltration, indicating a dose‐dependent anti‐inflammatory effect. Lastly, we assessed apoptosis using cleaved caspase‐3 staining, which demonstrated high levels of apoptosis after APAP exposure. Again, this effect was markedly diminished upon CMM‐98 treatment, consistent with reduced hepatocyte death (Figure 3G,H).

Since the mitochondrial isoform of OGG1 has been demonstrated to play significant roles in the protection against high levels of oxidative stress [17, 18, 19], we investigated mitochondrial morphology using ultrastructural examination by transmission electron microscopy. We observed that APAP caused profound mitochondrial swelling, with aspect ratios approaching 1, indicative of spherical transformation and structural collapse (Figure 3I,J). In contrast, CMM‐98 restored mitochondrial morphology, normalizing both mitochondrial area and long‐to‐short axis ratio toward physiological levels (Figure 3I,J).

Finally, we investigated whether this protective effect was attributable to enhanced liver structure regeneration. Employing a 70% partial hepatectomy model (Figure S3A), we surprisingly observed that Ki67 staining revealed no increase in hepatocyte proliferation following CMM‐116, CMM‐98, or TH10785 treatment. Rather, proliferation indices were modestly reduced in TH10785 and CMM‐98 groups (Figure S3B,C).

Together, these results indicate that CMM‐98 mitigates APAP‐induced acute liver failure in a murine model by accelerating oxidative DNA repair, suppressing oxidative stress, limiting inflammatory infiltration, and preserving mitochondrial structural integrity, thereby preventing hepatocyte apoptosis. Although OGG1 is present in both the nucleus and mitochondria, the present study does not distinguish the relative contribution of nuclear vs. mitochondrial DNA repair to these protective effects.

### 2.4 OGG1 Activation Protects Primary Human Hepatocytes From APAP Toxicity by OGG1 Toxicity

**FIGURE 4:**
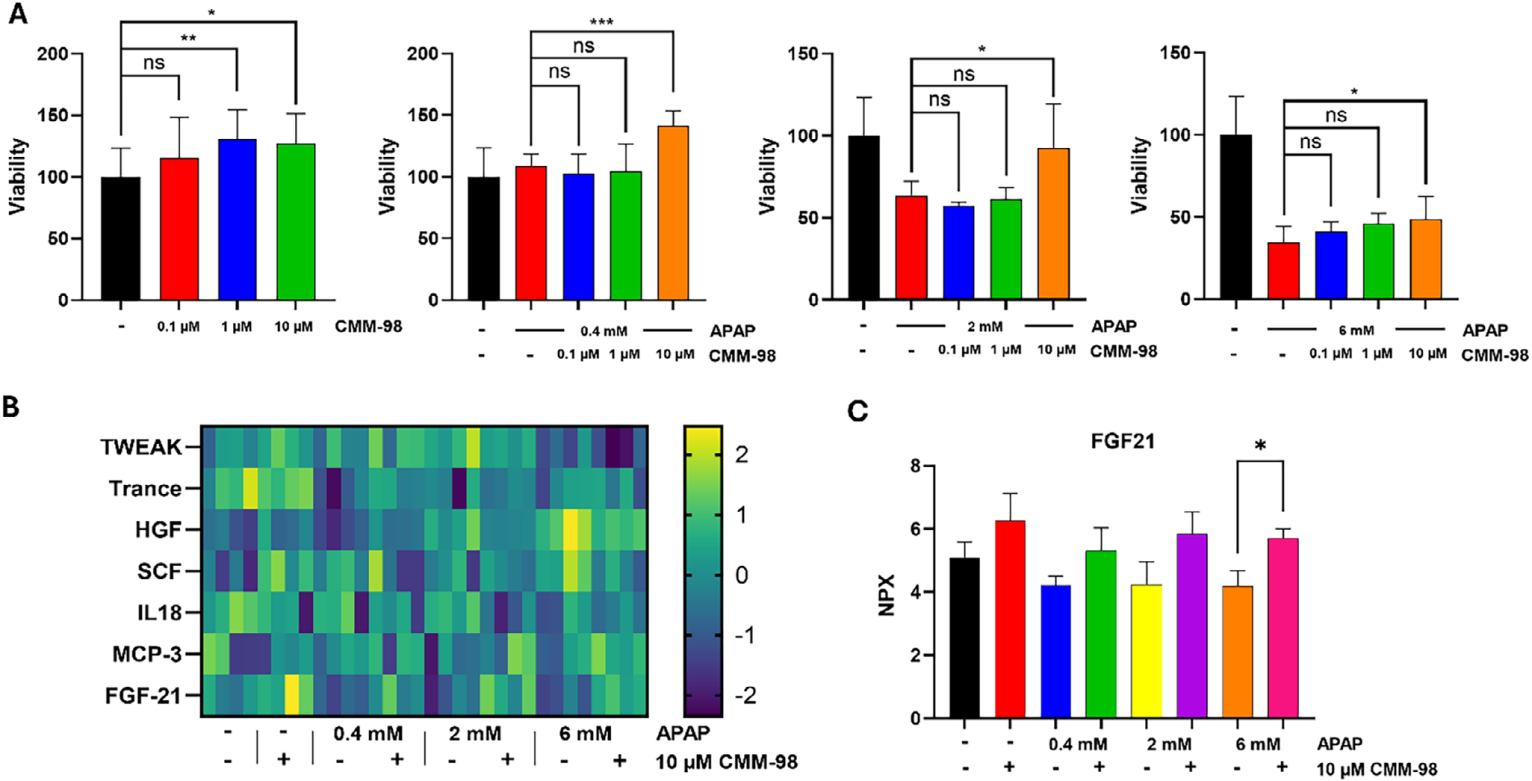
OGG1 ORCAs protect primary human hepatocytes from acetaminophen‐induced toxicity and induce adaptive stress signaling. (A) Primary human hepatocytes were exposed to increasing concentrations of acetaminophen (APAP; 0.4, 2, and 6 mM) in the presence or absence of CMM‐98 (0.1, 1, or 10 µM). Cellular viability was quantified by ATP‐based metabolic readout. CMM‐98 improved hepatocyte viability across APAP concentrations, including under conditions of severe toxicity. Data are presented as mean ± SD. Statistical significance was determined using Welch's *t*‐test. (B) Heatmap representation of Olink secretome profiling from primary human hepatocytes exposed to APAP and CMM‐98. Protein levels were Z‐score normalized per protein across all samples. Treatment with CMM‐98 induced selective remodeling of the hepatocyte stress secretome, including modulation of inflammatory mediators and induction of adaptive stress‐associated proteins. (C) Quantification of FGF21 levels measured by Olink NPX values across APAP concentrations ± 10 µM CMM‐98. CMM‐98 treatment was associated with increased FGF21 expression under acetaminophen‐induced stress conditions, consistent with activation of an adaptive hepatocyte stress response. Data are presented as mean ± standard deviation (SD) from biological replicates. Statistical significance was assessed using Welch's *t*‐test. ^*^ *p* < 0.05, ^**^ *p* < 0.01, ^***^ *p* < 0.001; ns, not significant.

To increase the translational relevance of our findings and account for species‐specific differences, we next assessed the efficacy of OGG1 ORCAs in organotypic 3D cultures of primary human hepatocytes. Using ATP as a proxy for mitochondrial integrity and cellular viability, we quantified APAP in vitro hepatotoxicity in the presence or absence of different ORCAs, including TH10785, CMM‐98, and CMM‐02 (Figure 4A and Figure S3) [33]. Three APAP concentrations were selected to represent low (0.4 mM), moderate (2 mM), and severe (6 mM) toxicity. While hepatocyte viability was largely unaffected at 0.4 mM APAP, exposure to 2 and 6 mM significantly reduced ATP levels, indicating substantial cellular stress and injury. Treatment with OGG1 ORCAs revealed compound‐specific effects. CMM‐98 significantly blunted APAP‐hepatotoxicity, including at 6 mM APAP, where cellular stress is most pronounced (Figure 4A). In contrast, CMM‐02 remained ineffective, and TH10785 displayed protective effects only at lower APAP concentrations but worsened viability at higher doses, suggesting context‐dependent toxicity (Figure S3).

To further characterize the cellular response, we performed protein secretome profiling using the Olink proximity extension assay (Figure 4B,C). This proteomic analysis revealed that APAP exposure induced a stress‐associated hepatocyte secretory program, including upregulation of chemokines and damage‐associated mediators. Treatment with CMM‐98 did not result in global suppression of this response but instead led to a selective remodeling of the secretome. Notably, CMM‐98 treatment was associated with an induction of FGF21 across APAP concentrations, particularly under conditions of moderate to severe stress, consistent with activation of an adaptive hepatocyte stress response. In parallel, levels of the damage‐associated cytokine were reduced under conditions of intermediate APAP toxicity, suggesting attenuation of damage‐driven inflammatory signaling.

Together, these findings indicate that pharmacological activation of OGG1 preserves hepatocyte viability under stress induced by acetaminophen and promotes a shift from damage‐associated signaling toward an adaptive, FGF21‐associated stress response in primary human hepatocytes.

## 3 Discussion

ALF represents severe manifestations of acute organ injury that lead to rapid hepatocyte loss with frequently fatal outcomes [1, 46]. Although the pathogenic role of ROS in toxin‐induced liver injury has been extensively documented, considerably less attention has been directed toward the cellular capacity to resolve the resulting oxidative DNA damage [11, 47, 48]. In this study, we demonstrate that modulation of the DNA glycosylase OGG1 through small‐molecule organocatalytic switches, called ORCAs, enhances oxidative DNA repair capacity, which can protect hepatocytes from acute toxic injury. Using the next‐generation compound CMM‐98, we show that chemical switching of OGG1 catalysis accelerates BER, reduces oxidative DNA lesions, preserves mitochondrial integrity, and improves survival in a murine model of acetaminophen‐induced ALF.

Our findings extend the emerging concept that DNA repair enzymes are not static housekeeping factors but rather dynamic catalytic systems whose activity profiles can be chemically reprogrammed [26]. Previous work demonstrated that ORCAs can alter the catalytic behavior of OGG1, enabling enhanced processing of repair intermediates and increased BER flux [15, 16, 31, 38, 49]. In the present study, we build upon this concept by introducing a new chemical scaffold that installs a robust *β*‐AP lyase activity in OGG1.

Exploring chemical space as guided by the 2‐ and 4‐positions in a pyrimidinyl‐core introduced previously [16, 28, 29, 31, 32], we were, among others, able to include a linker scaffold connecting a second aromatic ring to a previously untargeted distal pocket in OGG1. This addition to the reactive core significantly expanded molecular complexity and molecular weight as compared to earlier fragment‐like structures of ORCAs targeting OGG1. Structural analysis indicates that the analog CMM‐115 engages the OGG1 catalytic pocket through extended interactions involving known interactions of Asp268, Phe319, and Gly42, as well as now Gln315, resulting in enhanced ligand‐dependent rearrangement of the active‐site architecture compared to earlier ORCAs [16, 22, 31, 32, 34]. These structural observations are supported by mutational analysis demonstrating loss of activity in variants affecting key catalytic residues, such as Phe319Ala, Asp268Ala, and Cys253Tyr. This effect was retained, although reduced, in the Ser326Cys variant, which is distal to the active site [22, 35]. Together, these results indicate that CMM‐98 acts through direct active‐site engagement to modulate OGG1 catalytic outcome.

A central observation of our study is that CMM‐98 promotes an APE1‐dependent BER pathway, in contrast to the previously described largely PNKP1‐dependent mechanism associated with the first‐generation ORCA TH10785 [16, 21]. This distinction was evident in several orthogonal assays, including immunofluorescence analysis of APE1 recruitment, comet assay measurements of oxidative lesions, and dynamic quantification of AP sites using the UBER probe [41, 42]. The transient accumulation followed by rapid resolution of AP sites observed with nanomolar amounts of CMM‐98 suggests accelerated turnover of BER intermediates, consistent with increased repair flux rather than simple lesion suppression. These results support a model in which chemical switching of OGG1 using catalytic amounts of intervention alleviates a rate‐limiting step in BER under conditions of extreme oxidative stress.

Importantly, enhanced repair capacity translated into robust protection against acetaminophen‐induced liver failure in vivo. Pretreatment with CMM‐98 markedly improved survival in a lethal APAP challenge model and preserved liver function as reflected by reduced ALT and AST levels. Histopathological analyses further demonstrated attenuation of hepatocellular necrosis, reduced apoptotic signaling, and decreased inflammatory infiltration. These protective effects were accompanied by reduced ROS accumulation and improved mitochondrial morphology, suggesting that enhanced DNA repair capacity indirectly stabilizes mitochondrial function during acute oxidative collapse [17, 18, 19, 28, 50].

The preservation of mitochondrial structure observed in our study is particularly noteworthy, given the well‐established role of mitochondrial dysfunction in APAP‐induced hepatotoxicity [51, 52]. Mitochondrial swelling and fragmentation are hallmarks of oxidative damage and permeability transition during acute liver injury. Our ultrastructural analyses indicate that CMM‐98 restores mitochondrial elongation and aspect ratio toward physiological values, suggesting improved mitochondrial resilience. However, the mechanistic relationship between enhanced DNA repair and mitochondrial protection requires careful interpretation. Efficient repair of oxidative nuclear DNA lesions is expected to reduce persistent DNA damage signaling, ROS amplification, and cellular stress, thereby indirectly preserving mitochondrial homeostasis. In parallel, OGG1 is known to localize to mitochondria and participate in mitochondrial base excision repair [17, 18, 19]. Although our data demonstrate enhanced BER flux following CMM‐98 treatment, we did not directly distinguish nuclear from mitochondrial DNA repair. Therefore, the mitochondrial protection observed here likely reflects a combination of reduced oxidative stress resulting from enhanced BER and a potential contribution from mitochondrial OGG1‐mediated repair, which remains to be directly investigated [50].

In addition to preserving mitochondrial integrity, OGG1 activation was associated with a distinct shift in hepatocyte stress signaling in primary human hepatocytes. Secretome profiling revealed robust induction of FGF21 following treatment with CMM‐98. FGF21 is a well‐established hepatokine linked to metabolic adaptation and mitochondrial stress responses, and has been implicated in hepatoprotection in both preclinical and clinical settings [53, 54]. Its induction in this context suggests that enhanced BER enables hepatocytes to engage adaptive stress programs rather than progressing toward irreversible injury.

In parallel, we observed selective attenuation of damage‐associated inflammatory mediator IL18, consistent with reduced damage‐driven signaling [55, 56]. Notably, this effect did not reflect a global suppression of the hepatocyte secretory response, but rather a qualitative shift in signaling output. These findings support a model in which pharmacological activation of OGG1 promotes a controlled and adaptive response to acute toxic stress, linking enhanced DNA repair capacity to downstream metabolic and signaling adaptations in human hepatocytes.

Interestingly, the hepatoprotective effects observed in this study do not appear to arise from enhanced liver regeneration. In the partial hepatectomy model, ORCA treatment did not increase hepatocyte proliferation and instead showed a modest reduction in Ki67 staining. These results suggest that the primary effect of OGG1 modulation lies in improving intrinsic hepatocyte resilience rather than stimulating compensatory regeneration. This observation aligns with the concept that early hepatocyte survival during acute injury is a critical determinant of clinical outcome [57].

From a therapeutic perspective, the identification of DNA repair capacity as a modifiable determinant of acute liver injury has several implications. Current pharmacological approaches to ALF largely focus on upstream events such as detoxification, inflammation, or oxidative stress [58]. Our data suggest that targeting downstream damage resolution pathways may represent a complementary strategy. Enhancing BER flux during acute oxidative stress could allow hepatocytes to tolerate otherwise lethal DNA damage loads, thereby extending the therapeutic window during acute toxic exposure.

While APAP toxicity represents the most common cause of ALF in Western countries, it remains to be determined whether ORCA‐mediated DNA repair enhancement would confer protection in post‐exposure scenarios and in other forms of acute liver injury, including ischemia‐reperfusion injury, viral hepatitis, or immune‐mediated liver failure.

Together, our findings establish that chemical switching of DNA repair enzymes can enhance cellular tolerance to extreme oxidative stress. By converting OGG1 into an efficient AP‐site processing enzyme, ORCAs provide a strategy to accelerate BER under conditions in which repair capacity would otherwise become saturated. These results highlight DNA repair modulation as a potential therapeutic avenue for acute liver injury and possibly other oxidative stress‐driven pathologies.

An additional consideration for therapeutic development is the long‐term safety and selectivity of pharmacologically enhancing DNA repair. In the present study, CMM‐98 was administered only as a short‐term intervention during acute liver injury, where transient enhancement of BER is intended to restore endogenous repair capacity under conditions of overwhelming oxidative stress. Importantly, low amounts of CMM‐98 accelerate the turnover of OGG1‐mediated repair intermediates rather than introducing an alternative repair pathway or altering substrate specificity. Nevertheless, whether prolonged activation of BER could influence mutational landscapes or genome stability during chronic administration remains unknown. In addition, although CMM‐98 was identified as an OGG1‐ORCA and exhibited selectivity against representative DNA glycosylases and a wider kinase panel, its potential effects on components beyond BER were not investigated in the present study. Future biochemical, structural, and pharmacological studies will be important to determine whether CMM‐98 is highly selective for OGG1, further modulates additional DNA repair enzymes, or whether the rapid hepatic clearance generates an active metabolite.

## Author Contributions

**Zhenjun Zhao**: conceptualization, methodology, investigation, data curation, formal analysis, validation, supervision, funding acquisition, project administration, visualization, resources, Writing – review and editing. **Rahul Upadhyay**: investigation, methodology, formal analysis. **Alice Eddershaw**: methodology, validation, visualization. **Chenchen Wang**: methodology, validation, investigation. **Yudong Zhao**: methodology, validation, investigation. **Olov Wallner**: methodology, investigation, validation. **Jinhye Ryu**: methodology, investigation, validation. **Nayere Taebnia**: supervision, methodology, investigation, validation. **Heather Gildie**: methodology, investigation, validation. **Emma Scaletti‐Hutchinson**: methodology, investigation, validation. **Jonathan R. Davies**: methodology, investigation, validation. **Nicole Ziegler**: methodology, investigation, validation. **Andreas Krämer**: methodology, formal analysis. **Samantha C. Robinson**: methodology, formal analysis. **Marek Varga**: methodology, investigation, validation. **Karolina Singerova**: methodology, investigation, validation. **Zuzanna Szaruga**: methodology, investigation, validation. **Elisée Wiita**: methodology, validation, investigation. **İrşil Güneş**: methodology, investigation, validation. **Sheila S. David**: supervision, funding acquisition. **Stefan Knapp**: funding acquisition, supervision. **Aimo Kannt**: resources, writing – review and editing, supervision. **Miguel de Vega**: methodology, data curation, investigation, formal analysis, supervision, funding acquisition, writing – review and editing, resources. **Pål Stenmark**: supervision, resources. **Maurice Michel**: conceptualization, methodology, data curation, investigation, validation, formal analysis, supervision, funding acquisition, visualization, project administration, resources, writing – original draft, writing – review and editing.

## Funding

This work was funded by the Åke‐Olsson foundation for haematological research (2020‐00306, MM), a Novo Nordisk Pioneer Innovator Grant (NNF23OC0085944, MM), the Åke Wiberg Foundation (M23‐0043, M25‐0337 MM), Magnus Bergvalls Stiftelse (2025‐528, MM), the Maximon Longevity Foundation (MM), grant PID2023‐148757NB‐I00 funded by MCIU/AEI/10.13039/501100011033 (MdV), and Karolinska Institute Research Foundation Grant (2022‐01776, MM). PS was supported by grants from the Swedish Cancer Society (24 3848 Pj) and the Swedish Research Council (2022‐03681). VML acknowledges support from the ERC Consolidator Grant 3DMASH [101170408], the Swedish Research Council [2021‐02801, 2023–03015 and 2024‐03401], Cancerfonden [24‐3735Pj] and the Robert Bosch Foundation, Stuttgart, Germany. NT acknowledges support by Karolinska Institute Research Foundation Grant (2024‐03194) and Special grants for young scientists. SSD acknowledges support from the National Cancer Institute (CA067985). This project has received funding from the Innovative Medicines Initiative 2 Joint Undertaking (JU) under grant agreement No 875510 (MM, OW, EW, VML). The JU receives support from the European Union's Horizon 2020 research and innovation programme and EFPIA and Ontario Institute for Cancer Research, Royal Institution for the Advancement of Learning McGill University, Kungliga Tekniska Högskolan, Diamond Light Source Limited. This communication reflects the views of the authors, and the JU is not liable for any use that may be made of the information contained herein. NZ and AK were supported by the Fraunhofer Innovation Center TheraNova funded by the Fraunhofer Society and the Hessian Ministry of Science and Arts (HMWK).

## Ethical Approvals

The animal study in this project was approved by the Ethics Committee in Renji Hospital and the approval number is RJ2024‐101.

## Conflicts of Interest

VML is CEO and shareholder of HepaPredict AB, as well as co‐founder and shareholder of Shanghai Hepo Biotechnology Ltd. MM is a consultant to Novartis.

## Supporting information




**Supporting File**: advs77956‐sup‐0001‐SuppMat.docx.

## Data Availability

The data underlying this manuscript are available from the authors upon reasonable request.
