# Supplementary material for: Stimulation of OGG1 Enhances Oxidative DNA Damage Repair and Protects Against Acute Liver Failure by Acetaminophen
Source: Adv Sci (Weinh). 2026 Sep 27:e77956. Online ahead of print. doi: 10.1002/advs.77956 (PMC13616348; doi:10.1002/advs.77956)
Supplement: Supplementary file 1 [file ADVS-9999-e77956-s001.docx]

Supporting Information for:

**Stimulation of OGG1 Enhances Oxidative DNA Damage Repair and Protects Against Acute Liver Failure by Acetaminophen**

Zhenjun Zhao^1,#,*^, Rahul Upadhyay^2,3,#^, Alice Eddershaw^3,#^, Chenchen Wang^1,#^, Yudong Zhao^1,#^, Olov Wallner^2,#^, Jinhye Ryu^4^, Nayere Taebnia^4^, Heather Gildie^2^, Emma Scaletti-Hutchinson^5^, Jonathan R. Davies^5^, Nicole Ziegler^6^, Andreas Krämer^7^, Samantha C. Robinson^8^, Marek Varga^2^, Karolina Singerova^2^, Zuzanna Szaruga^2^, Elisée Wiita^2^, İrşil Güneş^3^, Sheila S. David^8^, Stefan Knapp^7^, Aimo Kannt^6,9^, Miguel de Vega^10^, Pål Stenmark^5^, Volker M Lauschke^4,11,12, 13, 14^, Maurice Michel^2,3*^

**Affiliations**

1. Department of Liver Surgery, Renji Hospital, School of Medicine, Shanghai Jiao Tong University, Shanghai, China.

2. Science for Life Laboratory, Department of Oncology-Pathology, Karolinska Institutet, Solna, Sweden.

3. Center for Molecular Medicine, Department of Oncology-Pathology, Karolinska Institute and Karolinska Hospital, Stockholm, Sweden.

4. Department of Physiology and Pharmacology, Karolinska Institutet, Stockholm, 17165 Sweden

5. Department of Biochemistry and Biophysics, Stockholm University, Stockholm, Sweden

6. Fraunhofer Institute for Translational Medicine and Pharmacology (ITMP), 60528, Frankfurt am Main, Germany

7. Institute of Pharmaceutical Chemistry & Structural Genomics Consortium (SGC), Goethe University, Frankfurt, Germany

8. Department of Chemistry and Chemistry and Chemical Biology Graduate Program, University of California, Davis, CA 95616 USA

9. Institute of Clinical Pharmacology, Goethe University, 60596 Frankfurt am Main, Germany

10. Centro de Biología Molecular Severo Ochoa (CSIC-UAM), Madrid, Spain

11. Center for Molecular Medicine, Karolinska Institutet and University Hospital, Stockholm, 17176 Sweden

12. Dr. Margarete Fischer-Bosch Institute of Clinical Pharmacology (IKP), 70376 Stuttgart, Germany

13. University of Tübingen, 72074 Tübingen, Germany

14. Department of Pharmacy, the Second Xiangya Hospital, Central South University, Changsha, 410011 China

**Supplementary Figure 1**

**Supplementary Figure 1.** Comparison of mouse OGG1-CMM-115 (blue) with human OGG1-TH10785 (orange): (A) Superposition of mOGG1-CMM-115 (blue) and human-TH10785 (orange, PDB ID: 7AYY) monomers. Ligands are depicted as sticks; C atoms are coloured blue (CMM-115) or orange (TH10785), N atoms dark blue and O atoms red. (B) Comparison of ligand binding between the structures. Amino acids which contribute to ligand positioning are depicted as thin sticks. The hydrogen bond network for human-TH10785 is indicated with dashed lines. Figure produced with PyMOL (version 3.0.4, Schrödinger).

**Supplementary Table 1: X-ray crystallography data collection and refinement statistics**

|  | mOGG1-CMM-115 |
| --- | --- |
| Data collection |  |
| PDB code | 9TM4 |
| Station | MAXIV-BioMAX |
| Space group | P2_1_2_1_2_1_ |
| Cell dimensions: |  |
| a, b, c (Å) | 81.8, 82.0, 169.7 |
| α, β, γ (°) | 90, 90, 90 |
| Resolution (Å) | 169.7-2.40 (2.48-2.40) |
| Total reflections | 616803 (61200) |
| Unique reflections | 45589 (4428) |
| *R*_merge_ | 10.0 (197.8) |
| *R*_pim_ | 4.0 (79.3) |
| CC_1/2_ (%) | 99.9 (59.8) |
| *I/*σ | 15.0 (1.4) |
| Completeness | 100 (100) |
| Redundancy | 13.5 (13.8) |
| Refinement |  |
| *R*_work_/*R*_free_ (%) | 23.6/29.1 |
| *B-*factors: |  |
| Protein (all atoms)^a^ | 57.4/81.3/90.9 |
| Ligand ^a^ | 44.7/63.9/66.0 |
| Water | 56.4 |
| R.m.s. deviations: |  |
| Bond lengths (Å) | 0.010 |
| Bond angles (°) | 1.49 |
| Ramachandran statistics: |  |
| Favoured (%) | 99.36 |
| Outliers (%) | 0.64 |

*Values in parentheses are for the highest-resolution shell. ^a^ Values for each monomer (A, B and C) of the asymmetric unit.*

**Supplementary Table 2:** Selectivity of CMM-98 for a panel of DNA glycosylases. The activity of DNA glycosylase enzymes SMUG1, TDG, UNG2, APE1 treated with 1, 0.125 or 0.0156 µM CMM-98 was compared to DMSO control to assess inhibition of the enzymes by the ORCA, using fluorescent EUbOPEN glycosylase protocols as below. 10 nM SMUG1, TDG or UNG2 coupled with 2.5 nM APE1 were assayed with 10 nM DNA containing U:G, and end-point RFU activity at was compared to that of the DMSO control. Endpoints were taken at 400 minutes for SMUG1, 600 mins for TDG, and 60 minutes for UNG2. APE1 alone was assessed at 2.5 nM with 10 nM AP site:G containing DNA, generated by pre-treatment of U:G containing DNA with UNG2 and activity compared to DMSO control at 60 minutes. MUTYH activity in the presence of CMM-98 (1, 0.125 and 0.0156 µM) was assessed using a gel-based adenine glycosylase assay.^[1]^ MUTYH (10 nM) was incubated with 10 nM 5′-[³²P]-labeled 30 bp duplex DNA containing a central OG:A mismatch (20 mM Tris-HCl, pH 7.6, 1 mM EDTA, 30 mM NaCl and 0.1 mg/mL BSA). Reactions were incubated at 37 °C for 60 minutes with reaction progression monitored using by denaturing PAGE and visualized by storage phosphor autoradiography. Activity for each CMM-98 concentration was normalized to the mean activity of the DMSO control and reported as percent activity relative to DMSO.

|  | **%activity to DMSO control** | | |
| --- | --- | --- | --- |
| **Protein ID** | **1 µM CMM-98** | **0.125 µM CMM-98** | **0.0156 µM CMM-98** |
| **SMUG1** | 79 | 79 | 85 |
| **TDG** | 83 | 94 | 94 |
| **UNG2** | 105 | 97 | 95 |
| **APE1** | 113 | 110 | 95 |
| **MUTYH** | 99 | 99 | 99 |

**Supplementary Table 3**: Results from the DSF-based selectivity screening against a curated kinase library and other typical off targets (specifically stated). The assay was performed as described in Materials and Methods using compound CMM-98.

| **Protein** | **native TM** | **native TM. Error** | **CMM-98 TM-shift** | **CMM-98 TM-shift Error** | **Reference Compound** | **Reference TM shift** |
| --- | --- | --- | --- | --- | --- | --- |
| STK39 | 45,0 | 1,9 | 2,7 | 0,2 | Staurosporine | 11,4 |
| STK4 | 47,1 | 0,3 | 2,4 | 0,1 | Staurosporine | 15,5 |
| MAP2K6 | 47,4 | 0,4 | 2,3 | 0,9 | Staurosporine | 11,3 |
| CAMK4 | 40,4 | 0,3 | 2,3 | 0,7 | Staurosporine | 8,2 |
| SRC | 48,0 | 1,5 | 1,5 | 0,0 | Staurosporine | 5,0 |
| MAP3K5 | 41,5 | 0,8 | 1,5 | 1,1 | Staurosporine | 15,5 |
| GPRK5 | 47,2 | 0,2 | 1,4 | 0,0 | Staurosporine | 5,9 |
| BMP2K | 36,7 | 0,6 | 1,3 | 0,4 | Staurosporine | 18,4 |
| CDC42BPA | 49,9 | 0,9 | 1,2 | 0,1 | Staurosporine | 0,0 |
| BRAF | 43,5 | 0,1 | 1,0 | 0,0 | Dabrafenib | 26,6 |
| CSNK2A1 | 47,9 | 0,3 | 1,0 | 0,3 | Similtasertib | 14,6 |
| MARK4 | 42,4 | 0,4 | 0,9 | 0,3 | Staurosporine | 14,3 |
| MAPK1 | 41,5 | 0,2 | 0,8 | 0,4 | GDC-0994 | 7,5 |
| BMX | 44,2 | 0,1 | 0,8 | 0,1 | Staurosporine | 6,8 |
| SRPK1 | 38,9 | 0,6 | 0,8 | 0,1 | Staurosporine | 7,0 |
| RIOK1 | 45,0 | 0,4 | 0,8 | 0,3 | n.d. | 0,0 |
| PLK4 | 46,6 | 0,1 | 0,7 | 0,0 | Staurosporine | 18,0 |
| CSNK1D | 47,1 | 0,2 | 0,7 | 0,5 | PF-670462 | 9,0 |
| CDKL1 | 54,6 | 1,0 | 0,7 | 0,9 | CEP-32496 | 6,6 |
| PIM3 | 51,6 | 0,2 | 0,6 | 0,1 | Staurosporine | 19,0 |
| VRK1A | 49,9 | 1,2 | 0,6 | 0,5 | MR1 | 4,5 |
| DAPK1 | 36,0 | 0,0 | 0,6 | 0,1 | Staurosporine | 9,0 |
| PCTK1 | 36,4 | 0,3 | 0,6 | 1,0 | Staurosporine | 9,0 |
| AAK1 | 47,8 | 0,0 | 0,6 | 0,0 | Staurosporine | 15,3 |
| MERTK | 51,4 | 0,0 | 0,5 | 0,2 | Staurosporine | 5,5 |
| MELK | 49,0 | 0,0 | 0,5 | 0,0 | Staurosporine | 13,2 |
| GSG2 | 54,6 | 0,1 | 0,5 | 0,0 | Staurosporine | 8,9 |
| DYRK2 | 47,1 | 0,0 | 0,4 | 0,1 | Staurosporine | 7,3 |
| CLK3 | 50,8 | 0,0 | 0,4 | 0,2 | CLK-T3 | 15,0 |
| PIM1 | 34,2 | 0,1 | 0,4 | 0,0 | Staurosporine | 11,9 |
| MAP2K1 | 52,7 | 0,1 | 0,4 | 0,2 | Staurosporine | 3,2 |
| MAPK13 | 33,2 | 0,0 | 0,3 | 0,1 | Doramapimod | 0,0 |
| BMPR2 | 51,6 | 0,1 | 0,3 | 0,0 | JG337 | 11,8 |
| PAK4 | 32,3 | 0,0 | 0,3 | 0,1 | Staurosporine | 12,2 |
| AURKB | 44,5 | 0,3 | 0,3 | 1,1 | Staurosporine | 8,0 |
| NEK2 | 46,8 | 0,1 | 0,3 | 0,5 | VN2010 | 9,7 |
| MAPK14 | 46,3 | 0,5 | 0,3 | 0,4 | Doramapimod | 19,8 |
| CASK | 46,9 | 0,3 | 0,3 | 0,0 | Staurosporine | 5,2 |
| EPHA2 | 41,1 | 0,6 | 0,2 | 1,2 | Staurosporine | 7,1 |
| ULK1A | 50,3 | 0,2 | 0,1 | 0,1 | Staurosporine | 12,0 |
| FGFR3A | 38,3 | 0,1 | 0,1 | 0,1 | Staurosporine | 11,7 |
| CSNK2A2 | 39,0 | 0,1 | 0,1 | 0,1 | Similtasertib | 15,7 |
| GAK | 44,4 | 0,1 | 0,1 | 0,0 | Staurosporine | 8,8 |
| AKT3 | 62,5 | 0,0 | 0,1 | 0,3 | Staurosporine | 7,0 |
| GSK3B | 42,7 | 0,3 | 0,1 | 0,8 | Staurosporine | 9,0 |
| CAMK1G | 45,2 | 0,1 | 0,1 | 1,5 | Staurosporine | 8,8 |
| TAF1 | 48,4 | 0,4 | 0,1 | 0,2 | Bromosporine | 7,4 |
| FES | 36,0 | 0,4 | 0,1 | 0,3 | Staurosporine | 7,5 |
| ULK3A | 46,6 | 0,7 | 0,0 | 0,4 | Staurosporine | 17,3 |
| STK10 | 46,6 | 0,1 | 0,0 | 0,3 | Staurosporine | 23,5 |
| STK6 | 43,2 | 0,7 | 0,0 | 0,2 | Staurosporine | 16,5 |
| STK38L | 43,4 | 0,1 | 0,0 | 0,5 | Staurosporine | 13,1 |
| MAPK10 | 40,5 | 0,7 | 0,0 | 0,4 | CEP-32496 | 8,8 |
| MARK3 | 47,5 | 0,4 | 0,0 | 0,3 | Staurosporine | 18,0 |
| RPS6KA1 | 51,1 | 0,5 | -0,1 | 0,1 | Staurosporine | 3,2 |
| EPHA7 | 52,7 | 0,3 | -0,1 | 0,1 | Staurosporine | 10,5 |
| CAMK2D | 46,5 | 0,1 | -0,1 | 0,1 | Staurosporine | 15,8 |
| EPHA5 | 39,6 | 0,1 | -0,1 | 0,2 | Staurosporine | 6,9 |
| PAK1 | 43,0 | 0,3 | -0,1 | 0,2 | Staurosporine | 7,3 |
| MAP2K7 | 53,7 | 0,4 | -0,1 | 0,6 | Staurosporine | 7,0 |
| NEK1 | 51,2 | 0,1 | -0,1 | 0,0 | n.d. | 0,0 |
| DCAMKL1 | 51,1 | 0,1 | -0,2 | 0,1 | Staurosporine | 11,4 |
| ABL1 | 57,5 | 0,0 | -0,2 | 0,1 | Staurosporine | 8,6 |
| TTKA | 51,0 | 0,2 | -0,2 | 0,2 | Staurosporine | 9,0 |
| OSR1 | 47,2 | 0,3 | -0,2 | 0,1 | Staurosporine | 5,7 |
| FGFR1 | 54,1 | 0,0 | -0,2 | 0,2 | Staurosporine | 5,7 |
| CLK1 | 49,2 | 0,2 | -0,2 | 0,9 | Staurosporine | 15,7 |
| EPHB3 | 47,8 | 0,1 | -0,3 | 0,1 | Staurosporine | 7,2 |
| CHEK2 | 39,4 | 0,4 | -0,4 | 0,5 | Staurosporine | 17,1 |
| STK17A | 52,2 | 0,0 | -0,4 | 0,2 | Staurosporine | 7,7 |
| NEK7 | 56,4 | 0,1 | -0,4 | 0,2 | VN2010 | 5,5 |
| CAMKK2 | 47,7 | 0,2 | -0,4 | 0,2 | Staurosporine | 23,5 |
| STK24 | 49,0 | 0,1 | -0,5 | 0,4 | Staurosporine | 5,5 |
| DMPK1 | 41,4 | 0,0 | -0,5 | 0,1 | Staurosporine | 9,3 |
| CDK2 | 46,5 | 0,3 | -0,5 | 0,5 | Staurosporine | 15,2 |
| STK3 | 60,1 | 0,0 | -0,7 | 0,9 | Staurosporine | 13,4 |
| MAPK8 | 54,4 | 0,3 | -0,9 | 1,8 | Staurosporine | 7,7 |
| MAP2K4 | 40,5 | 1,2 | -1,0 |  | Staurosporine | 10,5 |
| MAPK9 | 46,7 | 0,0 | -1,2 | 1,3 | SBI-0069279 | 8,6 |
| FGFR2 | 42,8 | 0,9 | -1,3 | 0,4 | Staurosporine | 8,3 |

**Supplementary Figure 2**

**Supplementary Figure 2:** (**A**) Overall survival of mice after APAP and OGG1 activator treatment. Mice were fasted for 12 hours and given 750mg/kg APAP i.p. 1 hour later, mice were given DMSO, 10mg/kg TH10785 or 8mg/kg CMM98 i.p. and the survival of mice were observed every 4 hours until 60 hours (n = 10 mice in each group). (**B**) Serum ALT and AST levels in mice after APAP and TH10785 or CMM-98 treatment. Mice were injected with 10mg/kg TH10785 or 4mg/kg CMM98 i.p. for 2 hours and given 250mg/kg APAP. 3 hours, 6 hours and 12 hours after APAP treatment, mice were sacrificed and blood serum and liver tissues were harvested (n=6 in each group). (**C-D**) Typical figures (C) and quantification (D) of gH2AX positive nuclear percentage in the liver tissues of mice after APAP and TH10785 or CMM-98 treatment, as described in (B). (**C-D**) Typical figures (C) and quantification (D) of gH2AX positive cell counts per field in mice. Mice were treated with DMSO, 10mg/kg TH10785 or 4mg/kg CMM-98 for 1 hours before treated with 250mg/kg APAP i.p. Mice were sacrificed 3, 6 or 12 hours post APAP treatment and liver samples were harvested for following IHC staining.

**Supplementary Figure 3**

**Supplementary Figure 3:** (**A**) Schematic figure showing the design of hepatectomy model in mice. Mice were injected with different compounds for 8 hours and performed hepatectomy. 70-80% of liver was resected and the remaining liver tissues were harvested after 24 hours. (**B-C**) Typical figures (**B**) and quantification (**C**) of Ki67 staining results in the liver tissues.

**Supplementary Figure 4**

**Supplementary Figure 4. Differential effects of OGG1 ORCAs on primary human hepatocyte viability under acetaminophen-induced stress.** Primary human hepatocytes were exposed to increasing concentrations of acetaminophen (APAP; 0.4, 2 and 6 mM) in the presence or absence of the indicated OGG1 ORCAs. Cellular viability was quantified using an ATP-based luminescence assay as a measure of mitochondrial function and metabolic fitness. While CMM-98 consistently improved hepatocyte viability across APAP concentrations (Figure 4 main text), TH10785 displayed a biphasic response with protective effects at lower concentrations and reduced viability at higher doses. CMM-02 showed limited protective activity. Data are presented as mean ± standard deviation (SD) from biological replicates. Statistical significance was assessed using Welch’s t-test. *P < 0.05, **P < 0.01, ***P < 0.001; ns, not significant.

**Material and methods**

**Mice**

All mice were purchased from Shanghai MODEL ORGANISMS (https://www.modelorg.com/) and raised in the SPF-level Laboratory Animal Room located in Renji Hospital, affiliated to Shanghai Jiao Tong University, School of Medicine. Male mice at 6-8 weeks old were used in the study and were fed a standard chow diet unless otherwise stated. All animal experiments were performed in accordance with the European Convention for the Protection of Vertebrate Animals used for Experimental and Other Scientific Purposes, and were approved by the Animal Care and Use Committee of Renji Hospital Affiliated with Shanghai Jiao Tong University, School of Medicine (Shanghai, China), the ethics protocol number is RJ2024-101C.

**Isolation of primary mouse hepatocytes**

Primary hepatocytes were isolated from mice using a two-step collagenase perfusion method. Mice were treated with the indicated compounds for 8 hours prior to hepatocyte isolation. Livers were perfused and digested using type IV collagenase, followed by mechanical dissociation. Isolated hepatocytes were seeded in DMEM and allowed to attach. Non-adherent or detached cells were removed by washing, and adherent primary hepatocytes were used for subsequent experiments, including immunofluorescence staining, modified comet assay, and AP site quantification.

**Reagents and antibodies**

TUNEL staining was performed using the Colorimetric TUNEL Apoptosis Assay Kit (Beyotime, C1098) according to manufacturer’s protocol. FpG enzyme was purchased from NEB (New England Biolabs, M0240L). UBER probed was synthesized in Scilifelab, Stockholm, Sweden. DHE probe was purchased from Beyotime (S0064S) according to manufacturer’s protocol. APE1i and PNKP1i were purchased here (Sigma: 62017, A8736).

| Target | Application | Company | No. |
| --- | --- | --- | --- |
| gH2AX | IF | Cell Signaling Technology | 80312 |
| KI67 | IHC | Cell Signaling Technology | 9129 |
| p-53BP1 | IF | Cell Signaling Technology | 88439 |
| APE1 | IF | Abcam | ab189474 |
| F4/80 | IHC | Abcam | ab6640 |
| Cleaved Caspase-3 | IHC | Cell Signaling Technology | 9661 |

**Modified comet assay**

A modified comet assay was performed to evaluate oxidative DNA damage in primary hepatocytes as previously described. Cells were treated with 200 μM hydrogen peroxide (H₂O₂) for 30 minutes and then allowed to recover in the presence or absence of the indicated compounds. To specifically detect oxidative DNA lesions, formamidopyrimidine DNA glycosylase (FpG) enzyme was applied prior to electrophoresis. Comet electrophoresis was performed for 40 minutes. DNA damage was quantified by measuring tail moments using the OpenComet plugin in ImageJ.

**Proteins and reagents**

Enzymes (APE1, UNG2, TDG, SMUG1, MUTYH) as well as OGG1 and mutant variants were produced as reported previously.^[1–3]^ 8-oxoG-containing oligonucleotide (see sequence below) was radiolabelled at the 5' end with [γ^32^P]-ATP (Perkin-Elmer Life Sciences) and T4 polynucleotide kinase (T4PNK) from New England Biolabs.

**Proteins and reagents**

Labelled nucleotide [g^32^P]-ATP (3000 Ci/mmol) was from Revvity. T4 polynucleotide kinase (T4PNK) and *Escherichia coli* uracil DNA glycosylase (UDG) were from New England Biolabs. hOGG1 was purified as described.^[3]^

**DNA substrates**

Oligonucleotides were from Integrated DNA Technologies. For clarity, sequences are shown in each figure. Where indicated, ssDNA oligonucleotides were radiolabelled at the 5’ end with [g^32^P]-ATP and T4PNK. The dsDNA molecules were obtained by annealing the indicated labeled oligonucleotide to the non-labeled complementary one (1:2 ratio) in the presence of 30 mM HEPES (pH 7.4) and 0.2 M NaCl and heating to 90 °C for 5 min before slowly cooling to room temperature.

**Electrophoretic Mobility Shift Assays (EMSA)**

The reaction was carried out in a final volume of 20 µL, by incubating increasing concentrations of *h*OGG1 (1.25, 2.5 and 5 nM) with 1 nM of the 34mer [^32^P]5’-labeled 8oxodG:C containing DNA substrate, in the presence of 30 mM Hepes, pH 7.4, 4% (vol/vol) glycerol, 16 mM NaCl, 0.01% Tween-20, and either 10% DMSO or 6 µM of the specified CMM-98 or CMM-116 compound. Samples were analyzed by native 6% PAGE. After gel electrophoresis, the mobility of free dsDNA and the *h*OGG1-DNA complex was detected by autoradiography.

***h*OGG1 activity on radiolabeled 8oxoG-containing DNA.**

The reaction was carried out in a final volume of 12.5 µL, by incubating 1 nM of the 34mer [^32^P]5’-labeled 8oxoG-containing substrate, with 10 nM *h*OGG1, in the presence of 30 mM HEPES pH 7.4, 4% (vol/vol) glycerol, 16 mM NaCl, 0.01% Tween-20 and either 10% DMSO or 6 µM of the indicated CMM-98 or CMM-116 compound. Samples were incubated at 37 ºC for the specified times. The reaction was stopped by adding 10 µL of formamide buffer (20 mM EDTA, 95% formamide, 0.05% bromophenol blue and 0.05% xylencyanol blue) and further heating for 3 min at 95 ºC. DNA products were analyzed by 7M urea-20% PAGE and autoradiography.

**AP lyase assay on radiolabeled AP-containing DNA**

The assay was performed in a final volume of 12.5 µL by incubating 1 nM of the indicated 34mer [^32^P]5’-labeled uridine-containing substrate previously treated with 0.2 U of UDG to obtain a natural AP site, with 1 nM *h*OGG1, in the presence of 30 mM HEPES pH 7.4, 4% (vol/vol) glycerol, 16 mM NaCl, 0.01% Tween-20 and either 10% DMSO or 6 µM of the indicated CMM-98 or CMM-116 compound. After incubation at 37 ºC for the indicated times, reactions were either stopped by adding 10 µL of formamide buffer and further heating for 3 min at 95 ºC. DNA products were analyzed by 7M urea-20% PAGE and autoradiography.

**Crystallization**

Purified mOGG1 (22 mg/mL) was pre-incubated with 6.25 mM CMM-115 and crystallized via sitting drop vapor diffusion in 0.12 M Alcohols, 0.1 M imidazole/MES pH 6.5, 50 % EDO_P8K (Morpheus Screen, Molecular Dimensions) at 18 °C. Protein crystals were fished without additional cryoprotectant and cryo-cooled in liquid nitrogen.

**Data collection, structure determination, and refinement**

X-ray diffraction data were collected at BioMAX (Lund, Sweden) equipped with an EIGER X 16M detector. A complete dataset was collected on a single crystal at 100 K. The dataset was processed and scaled with XDS^[4]^ and AIMLESS^[5]^ within the CCP4 suite^[6]^. Molecular replacement was performed using MOLREP^[7]^ using the structure of mouse OGG1 (PDB ID: 6G3Y) with all ligands and waters removed, as the search model. Several rounds of manual model building and refinement were performed using COOT^[8]^ and REFMAC5^[9]^ during which waters and ligands were incorporated into the structures. Data processing and refinement statistics are presented in Supplementary Table 1. The coordinates and structure factors for mOGG1-CMM-115 were deposited in the PDB under the accession code 9TM4.

**DSF-based screening against OGG1**

The assay was performed as previously described.^[10]^ Briefly, proteins were used at a concentration of 4 μM and were mixed with 200 µM compound in a buffer containing 20 mM HEPES, pH 7.5, and 500 mM NaCl. SYPRO Orange (5000×, Invitrogen) was added as a fluorescence probe (1 µl per mL). Subsequently, temperature-dependent protein unfolding profiles were measured using the QuantStudio™ 5 Realtime PCR machine (Thermo Fisher). Excitation and emission filters were set to 465 nm and 590 nm, respectively. The temperature was raised with a step rate of 3°C per minute. Data points were analysed with the internal software (Thermal Shift Software^TM^ Version 1.4, Thermo Fisher) using the Boltzmann equation to determine the inflection point of the transition curve.

**DSF-based selectivity screening against OGG1 and a curated kinase library.** The assay was performed as previously described.^[11,12]^ Briefly, protein kinase domains at a concentration of 2 μM were mixed with 10 μM compound in a buffer containing 20 mM HEPES, pH 7.5, and 500 mM NaCl. SYPRO Orange (5000×, Invitrogen) was added as a fluorescence probe (1 µl per mL). Subsequently, temperature-dependent protein unfolding profiles were measured using the QuantStudio™ 5 Realtime PCR machine (Thermo Fisher). Excitation and emission filters were set to 465 nm and 590 nm, respectively. The temperature was raised with a step rate of 3°C per minute. Data points were analysed with the internal software (Thermal Shift Software^TM^ Version 1.4, Thermo Fisher) using the Boltzmann equation to determine the inflection point of the transition curve.

**Biochemical assay (Fluorescence)**

Enzymes were assayed as reported in Visnes *et al.*^[2]^ and “EUbOPEN” protocols (<https://www.eubopen.org/protocols-reagents>) and adapted where mentioned. OGG1 (10 nM) activation on 10 nM substrate was monitored in a kinetic mode and a time resolved curve was obtained for each compound concentration in triplicates except where stated otherwise. Initial slopes were taken of the linear part of these curves to determine rates and kinetics. Fluorescence values of each compound concentration were normalised by the DMSO control values and calculated as % activation of the full turnover fluorescence of the APE (2 nM) control. The median activation concentration (AC_50_) for each compound was calculated from the % activation of all tested concentrations and refers to the compound concentration which activates the reaction to 50% of the substrate turnover reached in the assay control coupled to APE1 (2 nM) as described before.^[3]^

**Immunofluorescence**

Primary hepatocytes were fixed with paraformaldehyde and blocked with 3% bovine serum albumin for 1 hour at room temperature. Cells were incubated with primary antibodies against γH2AX, APE1, and 53BP1, followed by incubation with species-matched secondary antibodies from Abcam. Fluorescence images were acquired using a Leica confocal microscope. Quantification of foci number, fluorescence intensity, and colocalization was performed using CellProfiler.

**Electron microscopy and mitochondrial analysis**

Transmission electron microscopy was performed by Servicebio (<https://www.service-bio.com/>) to examine mitochondrial ultrastructure in both primary hepatocytes and liver tissues. Samples were fixed with glutaraldehyde and processed according to standard protocols. Mitochondrial morphology was analyzed using ImageJ. The mitochondrial aspect ratio was defined as the ratio of the long axis to the short axis. For cell-based analyses, at least 20 mitochondria per cell were measured, and the average aspect ratio per cell was calculated. For tissue samples, mitochondria were analyzed from multiple fields per mouse. Mitochondrial density and mitochondrial area were quantified where indicated.

**In vivo models**

To establish the acetaminophen (APAP)-induced acute liver injury model, mice were fasted for 15 hours and pretreated with the indicated compounds. Mice subsequently received intraperitoneal injection of APAP at doses specified in the figure legends (200 mg/kg or 750 mg/kg). Animals were monitored for survival or sacrificed at the indicated time points for tissue and serum collection. Inhalation anesthesia was used where required.

For the partial hepatectomy model, mice were injected with the indicated compounds for 8 hours prior to surgery. Under inhalation anesthesia, approximately 70-80% of the liver was surgically resected. The remaining liver tissues were harvested 24 hours after hepatectomy for downstream analyses.

**Immunoblotting**

Liver tissues were collected at the indicated time points and lysed using RIPA buffer. Protein lysates were separated by SDS-PAGE and subjected to immunoblotting using antibodies against DNA damage-related markers. β-actin (ACTB) was used as a loading control. Immunoblot data are presented as representative images without quantitative densitometric analysis.

**In vivo ROS detection using DHE probe**

Reactive oxygen species levels in mouse livers were assessed using the dihydroethidium (DHE) probe. Twenty-four hours after APAP treatment, mice were intravenously injected with 100 μL of 5 mM DHE solution. Fifteen minutes after injection, mice were sacrificed, and liver tissues were harvested and embedded in OCT compound. Cryosections were prepared, and DHE fluorescence signals were imaged and quantified.

**Quantitative real-time PCR**

Total RNA was extracted from liver tissues and reverse-transcribed into complementary DNA. Quantitative real-time PCR was performed to assess gene expression levels. Expression values were normalized to ACTB as the internal control and calculated using the comparative Ct method. Primer sequences were listed in supplementary table.

**Serum ALT and AST measurement**

Blood samples were collected from mice 24 hours after APAP treatment. Serum alanine aminotransferase (ALT) and aspartate aminotransferase (AST) levels were measured using commercial assay kits from Beyotime Biotechnology according to the manufacturer’s instructions.

**Histology and immunohistochemistry**

Hematoxylin and eosin staining and immunohistochemistry analyses were performed by Servicebio. Liver tissues were fixed, embedded, sectioned, and processed according to standard histological procedures. Immunohistochemistry included TUNEL, F4/80, and Caspase 3 staining to assess apoptosis and inflammatory responses. Imaging and quantification were performed following the service provider’s standardized protocols.

**Liver spheroid culture**

Liver spheroid culture was performed as previously described^[13]^. Briefly, 1500 cryopreserved primary human hepatocytes (PHHs; BioIVT, USA) and 300 non-parenchymal cells (NPCs; LifeNetHealth, USA) were seeded per well into 96-well ultra-low attachment plates (Corning, USA). Experiments with patient-derived cells were approved by the Swedish Ethics Review Authority (Etikprövningsmyndigheten**)** under permit number 2024-05808-01. Cells were seeded in culture media (William’s E medium containing 11 mM glucose, 100 nM dexamethasone, 2 mM L-glutamine, 10 μg/mL insulin, 5.5 μg/mL transferrin, 6.7 ng/mL selenite, 100 U/mL penicillin, 100 μg/mL streptomycin) supplemented with 10% fetal bovine serum (FBS). After spheroid formation, FBS was phased out, and spheroids were maintained in culture media with different concentrations of APAP, TH10785, CMM-98, and CMM-02 as indicated. The exposure medium was exchanged with fresh medium every 2-3 days. After 7 days of treatment, spheroids were collected for cell viability assay. For Olink analyses, PHHs and NPCs were co-cultured at the same 5:1 ratio, with a total of 48,000 viable cells per well, into a 96-well Elplasia plate (Corning). Following spheroid formation, spheroids were treated with APAP at 0, 0.4, 2 or 6 mM, either alone or in combination with 10 μM CMM-98. After 72 hours of treatment, culture supernatants were collected.

**Cell viability**

Cell viability was evaluated using the CellTiter-Glo cell viability assay kit (Promega, USA). Individual spheroids in 25 μL of medium were transferred to wells of a white flat-bottom 96-well plate (Corning, USA). An equal volume of CellTiter-Glo reagent was added to each well and mixed thoroughly by brief agitation. The plate was incubated at 37°C for 20 minutes, and luminescence was measured using a microplate reader.

**Olink proteomics**

Targeted proteomic profiling of tumor samples was performed with proximity extension assay (PEA) technology using the Olink Target 96 Inflammation panel (Olink®, Uppsala, Sweden).

Analyses were performed following the manufacturer’s instructions. In short, 1µl of sample was incubated overnight with oligonucleotide-labelled antibody pairs of 92 proteins being covered in the panel. DNA duplexes were extended and afterwards amplified by PCR. Quantification was performed by high throughput qRT-PCR carried out on an integrated fluidic circuit chip (Standard BioTools, Boston, MA) on an Olink^®^ Signature Q100 instrument. Data was extracted and Ct values were normalized based on internal and external controls using the NPX Signature software (version 2.2.1). From those, normalized log2-scaled protein expression (NPX) values were calculated. Quality control parameters were applied as recommended by Olink.

**General Information Chemical Synthesis**

All reagents and solvents were purchased from commercial vendors and used without further purification. Unless otherwise stated, reactions were performed without care to exclude air or moisture. Analytical thin-layer chromatography was performed on silica gel 60 Å F-254 plates (E. Merck) and visualized under a UV lamp. Flash column chromatography was performed in a Biotage® SP1 MPLC system using Fisher Chemical silica gel 60 Å. ^1^H and ^13^C NMR spectra were recorded on Bruker DRX-400 MHz or Bruker Ascend 400 MHz spectrometers. Chemical shifts are expressed in parts per million (ppm) and referenced to the residual solvent peak (^1^H: δ 7.26 for CDCl3, δ 2.50 for DMSO-*d_6_*, δ 3.31 for methanol-*d_4_*; ^13^C: δ 29.84 for CDCl3, δ 39.52 for DMSO-*d_6_*, δ 49.00 for methanol-*d_4_*). Analytical LC-MS were performed either on an Agilent MSD mass spectrometer connected to an Agilent 1100 system or an Agilent 1260 infinity II with a G6125B mass spectrometer. Columns used were ACE 3 C8 (50 x 3.0 mm); H_2_O (+ 0.1% TFA) and MeCN were used as mobile phases at a flow rate of 1 mL/min, or Xterra MSC18 (50 x 3.0 mm) column where H_2_O (10 mM NH_4_HCO_3_; pH = 10) and MeCN were used as mobile phases at a flow rate of 1 mL/min. For LC-MS, detection was made by UV and MS (ESI+). Preparative LC was performed on a Gilson system using Waters C18 OBD 5 µm column (30 x 75 mm) with water buffer (50 mM NH_4_HCO_3_ at pH 10) and MeCN as mobile phases using a flow rate of 45 mL/min or ACE 5 C8 (100 x 21.2 mm) column with water buffer (0.1 % TFA) and MeCN as mobile phases using a flow rate of 30 mL/min. All final compounds were assessed to be >95% pure by LC-MS analysis. Compounds in our nucleobase/nucleoside/nucleotide and prior art screening set were not resynthesized and no characterization was performed. Compounds available through NCI DTP were not resynthesized and no characterization was performed. High-resolution mass spectra (HRMS) were measured with EI or ESI ionization. A chromatographic purification was performed before each measurement. The Thermo Q-Exactive plus device for ESI-mass spectra was coupled to a binary UHPLC system. For EI-measurement, a GC-system was coupled to the Thermo Q-Exactive GC device.

**Experimental Procedures and Spectral Data**

**6-methyl-N4-phenyl-N2-(2-(pyridin-3-ylamino)ethyl)pyrimidine-2,4-diamine (CMM-98)**

**Step 1:** A 50 mL flask was charged with 2,4-dichloro-6-methylpyrimidine **1** (20 mmol), methanol (15 mL), and triethylamine (20 mmol). Aniline **2** (20 mmol) was then added, and the reaction mixture was stirred at room temperature for 72 h. The crude product was purified by silica gel chromatography using 0-50% ethyl acetate in isohexane. Some colored impurities co-eluted. The product was further purified by recrystallization from ethyl acetate/isohexane to afford compound **6**. **Yield:** 64% as white solid.

**Step 2:** In a vial, 3-iodopyridine **4** (205 mg, 1.0 mmol), ethane-1,2-diamine **5** (0.20 mL, 3.0 mmol), CuCl (10 mg, 0.10 mmol), and powdered KOH (112 mg, 2.0 mmol) were combined and stirred at room temperature for 16 h. LCMS analysis indicated complete conversion. The reaction mixture was extracted with ethyl acetate and concentrated under reduced pressure. The crude product **6** was used directly in the next step without further purification.

**Step 3:** In a reaction vial, the compound **3** (50 mg, 0.227 mmol) and compound **6** (32 mg, 0.227 mmol) were combined with NMP (1 mL) and added potassium carbonate (95 mg, 0.682 mmol). The mixture was stirred at 140 ^o^C for 24 h. After cooling to room temperature, water was added to the reaction mixture and extracted with DCM 3 times. The combined organic layer was concentrated under reduced pressure, and the crude product was purified by silica gel chromatography to afford the desired compound **7**. **Yield:** 62%, as a brown solid. **^1^H NMR (400 MHz, DMSO-*d*_6_):** *δ* 9.06 (s, 1H), 7.98 (s, 1H), 7.74 (dd, *J* = 4.4, 1.6 Hz, 1H), 7.68 (d, *J* = 8.1 Hz, 2H), 7.24 (t, 8.4, 2H), 7.07 - 6.90 (m, 4H), 6.75 (s, 1H), 5.98 (s, 1H), 5.90 (s, 1H), 3.45 (q, *J* = 6.6 Hz, 2H), 3.25 - 3.19 (m, 2H), 2.13 (s, 3H). **^13^C NMR (176 MHz, DMSO-*d*_6_+H_2_SO_4_):** *δ* 161.3, 154.6, 153.3, 147.5, 138.0, 129.1, 128.3, 127.5, 124.8, 124.4, 121.4, 97.4, 41.5, 18.6. **HRMS (ESI):** m/z calc′d for C_18_H_20_N_6_ [M+H]+: 321.1822, found: 321.1820.

**(S)-2-((4-methyl-6-(phenylamino)pyrimidin-2-yl)amino)-N-phenylpropanamide (CMM-115)**

**Step 1:** In a vial, a mixture of 2,4-dichloro-6-methyl-pyrimidine (29 mg, 0.18 mmol), *L*-Alanine anilide (29 mg, 0.18 mmol), and DIPEA (0.040 mL, 0.23 mmol) was stirred in ethanol (0.50 mL) at 50 °C for 16 h. The mixture was then poured into NaHCO_3_ and extracted with DCM 3 times. The combined organic layer was concentrated under reduced pressure, and the crude product was purified by silica gel chromatography to afford the desired compound **9**. **Yield:** 19%.

**Step 2:** In a vial, a mixture of Compound **9** (10 mg, 0.034 mmol), aniline **2** (0.005 mL, 0.050 mmol), and DIPEA (0.009 mL, 0.050 mmol) was stirred in 2-propanol (1 mL) at reflux for 16 h. The crude material was purified by preparative LC (0.1% TFA in water/MeCN), and was isolated as the TFA salt. **Yield:** 37%, as a white solid. **^1^H NMR (400 MHz, DMSO-*d*_6_+H_2_SO_4_):** *δ* 10.54 (s, 1H), 10.22 (s, 1H), 8.31 (s, 1H), 7.57 (t, *J* = 9.1 Hz, 4H), 7.23 (t, *J* = 8.2 Hz, 2H), 7.13 (t, *J* = 8.0 Hz, 2H), 6.98 (t, *J* = 8.0 Hz, 2H), 6.22 (s, 1H), 4.48 - 4.44 (m, 1H), 2.23 (s, 3H), 1.43 (d, *J* = 5.5 Hz, 3H). **^13^C NMR (176 MHz, DMSO-*d*_6_+H_2_SO_4_):** *δ* 170.7, 161.4, 154.1, 153.4, 139.3, 138.2, 129.3, 129.2, 124.8, 123.9, 121.4, 119.7, 98.0, 51.7, 18.8, 18.3. **LC-MS:** m/z calc′d for C_20_H_21_N_5_O [M+H]+: 348.1, found: 348.1.

**N2,6-dimethyl-N4-phenylpyrimidine-2,4-diamine (34, CMM-116)**

**Step 1:** A 50 mL flask was charged with 4-chloro-6-methylpyrimidin-2-amine **29** (200 mg, 1.393 mmol) was dissolved in THF (8 mL, 40 V) and cooled it to 0 ^o^C and added NaH (185 mg, 6.965 mmol) and stirred at RT for 10 min. After this methyl iodide **31** (0.192 mL, 2.786 mmol) was added, and the reaction mixture was stirred at room temperature for 18 h. After this, the reaction mixture was quenched by adding water and extracted by DCM 3 times. The combined organic layer was concentrated under reduced pressure, and the crude product was purified by silica gel chromatography to afford compound **32** (**Yield:** 48%) and 33 (**Yield:** 36%).

**Step 2:** In a reaction vial, the compound **32** (50 mg, 0.317 mmol) and compound **2** (29.5 mg, 0.317 mmol) were combined with ethanol (2 mL, 40 V) and added a drop of conc. HCl. The mixture was stirred at 80 ^o^C for 2 h. After cooling to room temperature, saturated aq. NaHCO_3_ was added to the reaction mixture and extracted with DCM 3 times. The combined organic layer was concentrated under reduced pressure, and the crude product was purified by silica gel chromatography to afford the desired compound **34**. **Yield:** 74%, as an off white solid. **^1^H NMR (400 MHz, DMSO-*d*_6_):** *δ* 8.99 (s, 1H), 7.71 (d, *J* = 7.4 Hz, 2H), 7.27 - 7.23 (m, 2H), 6.91 (t, *J* = 7.4 Hz, 1H), 6.57 (s, 1H), 5.86 (s, 1H), 2.79 (d, *J* = 4.8 Hz, 3H), 2.10 (s, 3H). **LC-MS:** m/z calc′d for C_12_H_14_N_4_ [M+H]+: 215.1, found: 215.1.

**6-methyl-N4-phenyl-N2-(2-(pyridin-3-ylamino)ethyl)pyrimidine-2,4-diamine (CMM-98)**

**^1^H NMR**

**^13^C NMR**

**(S)-2-((4-methyl-6-(phenylamino)pyrimidin-2-yl)amino)-N-phenylpropanamide (CMM-115)**

**^1^H NMR**

**^13^C NMR**

**N2,6-dimethyl-N4-phenylpyrimidine-2,4-diamine (CMM-116)**

**^1^H NMR**

**Statistical Analysis**

Data are presented as mean ± standard deviation (SD), unless otherwise indicated in the corresponding figure legends. Individual biological replicates were included in all statistical analyses.

Statistical analyses were performed using two-sided tests with an α level of 0.05. Comparisons between two independent groups were performed using Welch's unpaired t-test when unequal variances were assumed. Comparisons involving more than two groups were analyzed using one-way or two-way analysis of variance (ANOVA), as appropriate, followed by Tukey's multiple-comparison test for post hoc analysis. Survival curves were analyzed using the Kaplan-Meier method and compared using the log-rank (Mantel-Cox) test. The assumptions of parametric testing, including independence of observations and approximate normal distribution of the data, were considered when selecting statistical tests. Exact statistical tests used for individual experiments are specified in the corresponding figure legends.

Statistical analyses were performed using GraphPad Prism (version 9, GraphPad Software, San Diego, CA, USA). Heatmap generation and Z-score normalization were performed in R (version 4.3). Image quantification was performed using ImageJ/Fiji and Cellprofiler where applicable. Statistical significance was defined as *P* < 0.05.

Prior to statistical analysis, raw data were inspected for completeness and consistency. For Olink proteomic analysis, normalized protein expression (NPX) values were used as provided by the manufacturer, and protein abundance values displayed in heatmaps were further transformed to Z-scores for visualization. No additional data transformation was applied unless otherwise specified. Images used for quantitative analyses were processed using identical acquisition and analysis parameters for all experimental groups.

No statistical methods were used to predetermine sample size. Sample sizes (n) for each experiment are indicated in the corresponding figure legends and represent independent biological replicates, individual animals, or independent experiments as appropriate.

**References:**

[1]	C. H. Trasviña-Arenas, U. C. Dissanayake, N. Tamayo, M. Hashemian, W. J. Lin, M. Demir, N. Hoyos-Gonzalez, A. J. Fisher, G. A. Cisneros, M. P. Horvath, S. S. David, “Structure of human MUTYH and functional profiling of cancer-associated variants reveal an allosteric network between its [4Fe-4S] cluster cofactor and active site required for DNA repair” *Nat Commun* **2025**, *16*, 3596.

[2]	T. Visnes, A. Cázares-Körner, W. Hao, O. Wallner, G. Masuyer, O. Loseva, O. Mortusewicz, E. Wiita, A. Sarno, A. Manoilov, J. Astorga-Wells, A.-S. Jemth, L. Pan, K. Sanjiv, S. Karsten, C. Gokturk, M. Grube, E. J. Homan, B. M. F. Hanna, C. B. J. Paulin, T. Pham, A. Rasti, U. W. Berglund, C. von Nicolai, C. Benitez-Buelga, T. Koolmeister, D. Ivanic, P. Iliev, M. Scobie, H. E. Krokan, P. Baranczewski, P. Artursson, M. Altun, A. J. Jensen, C. Kalderén, X. Ba, R. A. Zubarev, P. Stenmark, I. Boldogh, T. Helleday, “Small-molecule inhibitor of OGG1 suppresses proinflammatory gene expression and inflammation” *Science* **2018**, *362*, 834–839.

[3]	M. Michel, C. Benítez-Buelga, P. A. Calvo, B. M. F. Hanna, O. Mortusewicz, G. Masuyer, J. Davies, O. Wallner, K. Sanjiv, J. J. Albers, S. Castañeda-Zegarra, A.-S. Jemth, T. Visnes, A. Sastre-Perona, A. N. Danda, E. J. Homan, K. Marimuthu, Z. Zhenjun, C. N. Chi, A. Sarno, E. Wiita, C. von Nicolai, A. J. Komor, V. Rajagopal, S. Müller, E. C. Hank, M. Varga, E. R. Scaletti, M. Pandey, S. Karsten, H. Haslene-Hox, S. Loevenich, P. Marttila, A. Rasti, K. Mamonov, F. Ortis, F. Schömberg, O. Loseva, J. Stewart, N. D’Arcy-Evans, T. Koolmeister, M. Henriksson, D. Michel, A. de Ory, L. Acero, O. Calvete, M. Scobie, C. Hertweck, I. Vilotijevic, C. Kalderén, A. Osorio, R. Perona, A. Stolz, P. Stenmark, U. W. Berglund, M. de Vega, T. Helleday, “Small-molecule activation of OGG1 increases oxidative DNA damage repair by gaining a new function” *Science* **2022**, *376*, 1471–1476.

[4]	W. Kabsch, “XDS” *Acta Crystallogr D Biol Crystallogr* **2010**, *66*, 125–132.

[5]	P. Evans, “Scaling and assessment of data quality” *Acta Crystallogr D Biol Crystallogr* **2006**, *62*, 72–82.

[6]	Collaborative Computational Project, Number 4, “The CCP4 suite: programs for protein crystallography” *Acta Crystallogr D Biol Crystallogr* **1994**, *50*, 760–763.

[7]	A. Vagin, A. Teplyakov, “MOLREP: an Automated Program for Molecular Replacement” *Journal of Applied Crystallography* **1997**, *30*, 1022–1025.

[8]	P. Emsley, K. Cowtan, “Coot: model-building tools for molecular graphics” *Acta Crystallogr D Biol Crystallogr* **2004**, *60*, 2126–2132.

[9]	G. N. Murshudov, P. Skubák, A. A. Lebedev, N. S. Pannu, R. A. Steiner, R. A. Nicholls, M. D. Winn, F. Long, A. A. Vagin, “REFMAC5 for the refinement of macromolecular crystal structures” *Acta Cryst D* **2011**, *67*, 355–367.

[10]	M. Michel, T. Visnes, E. J. Homan, B. Seashore-Ludlow, M. Hedenström, E. Wiita, K. Vallin, C. B. J. Paulin, J. Zhang, O. Wallner, M. Scobie, A. Schmidt, A. Jenmalm-Jensen, U. Warpman Berglund, T. Helleday, “Computational and Experimental Druggability Assessment of Human DNA Glycosylases” *ACS Omega* **2019**, *4*, 11642–11656.

[11]	A. Krämer, C. G. Kurz, B.-T. Berger, I. E. Celik, A. Tjaden, F. A. Greco, S. Knapp, T. Hanke, “Optimization of pyrazolo[1,5-a]pyrimidines lead to the identification of a highly selective casein kinase 2 inhibitor” *Eur J Med Chem* **2020**, *208*, 112770.

[12]	O. Fedorov, F. H. Niesen, S. Knapp, “Kinase inhibitor selectivity profiling using differential scanning fluorimetry” *Methods Mol Biol* **2012**, *795*, 109–118.

[13]	C. C. Bell, D. F. G. Hendriks, S. M. L. Moro, E. Ellis, J. Walsh, A. Renblom, L. Fredriksson Puigvert, A. C. A. Dankers, F. Jacobs, J. Snoeys, R. L. Sison-Young, R. E. Jenkins, Å. Nordling, S. Mkrtchian, B. K. Park, N. R. Kitteringham, C. E. P. Goldring, V. M. Lauschke, M. Ingelman-Sundberg, “Characterization of primary human hepatocyte spheroids as a model system for drug-induced liver injury, liver function and disease” *Sci Rep* **2016**, *6*, 25187.
